# Engineering the Thermometric Response of Dinuclear Eu^III^ Complexes via Terminal Ligand-Induced Nonradiative Processes

**DOI:** 10.1021/acsomega.6c00433

**Published:** 2026-04-16

**Authors:** Ariane C. F. Beltrame, Rodolpho A. N. Silva, Sergio A. M. Lima, Luciano Marchiò, Matteo Melegari, Flavia Artizzu, Airton G. Bispo-Jr, Ana M. Pires

**Affiliations:** † School of Technology and Sciences, São Paulo State University (Unesp), Presidente Prudente, SP 19060-900, Brazil; ‡ Institute of Biosciences, Humanities and Exact Sciences, São Paulo State University (Unesp), São José do Rio Preto, SP 15054-000, Brazil; § Department of Sustainable Development and Ecological Transition, 19050 University of Piemonte Orientale “Amedeo Avogadro”, Piazza Sant’Eusebio 5, 13100 Vercelli, Italy; ∥ Department of Chemistry, Life Sciences and Environmental Sustainability, 9370 University of Parma, Parco Area delle Scienze 17/A, 43124 Parma, Italy; ⊥ Institute of Chemistry, 28133 University of São Paulo (USP), Lineu Prestes Street, 748, São Paulo 05508-900, Brazil

## Abstract

Luminescent temperature probes are powerful tools for monitoring various physical and chemical processes. Eu^III^ complexes are particularly attractive due to their bright luminescence and temperature sensitivity of the ^5^D_0_ excited-level lifetime. Since this thermal response is linked to nonradiative deactivation pathways, controlling these processes is key to tune thermometric behavior. Herein, we investigate how different terminal ligands modulate nonradiative deactivation and, consequently, the thermal luminescence response of dinuclear Eu^III^ complexes. As proof-of-concept, 1,3-diphenyl-1,3-propanedionate (dbm^–^) or 4,4,4-trifluoro-1-phenyl-1,3-butanedionate (btfa^–^) were employed as terminal ligands and 2,2′-bipyrimidine (bpm) as the bridge ligand to synthesize [Eu_2_(bpm)­(dbm)_6_] (**1**) and [Eu_2_(bpm)­(btfa)_6_] (**2**) complexes. The nature of the β-diketone impacts the crystal system, coordination geometry, and electronic structure. **1** presents coordination polyhedra described by a distorted D_2d_ point group while **2** displays a distorted D_4d_ coordination environment with more packed structure due to prominent F···F and H-bonding interactions. Both complexes present bright luminescence upon ultraviolet excitation, assigned to Eu^III 5^D_0_ → ^7^F_0–4_ transitions. However, the greater number of C–H bonds in dbm^–^ and less compact structure in **1** promote faster nonradiative decay and a lower activation energy for thermal quenching of luminescence. The terminal ligand also influences the S_1_ and T_1_ state energies and thus the intermolecular energy transfer as well as back energy transfer (BET) processes. Consequently, the thermometric performance using the ^5^D_0_ excited-level lifetime as the thermometric parameter is tuned by nonradiative contributions induced by the terminal ligand: **1** operates between 270 and 420 K, while **2** works from 300 to 440 K, with maximum relative sensitivities of 3.4% K^–1^ (370 K) and 3.6% K^–1^ (410 K), respectively. These findings demonstrate how ligand scaffolds enable fine-tuning of structure–property relationships for temperature-responsive luminescent materials.

## Graphical abstract

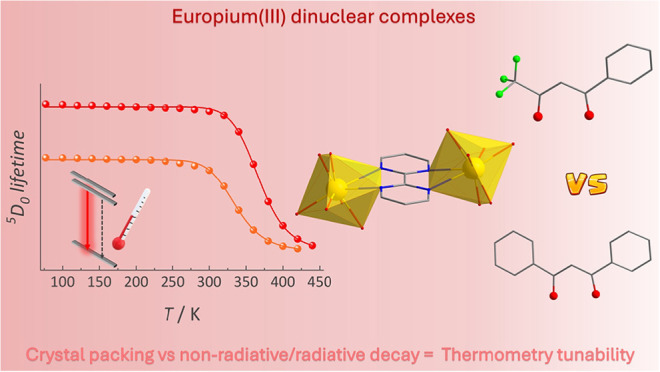

## Introduction

Precise temperature monitoring is critical in fields such as catalysis,^1,2^ microelectronics,^3^ and biomedical diagnostics,^4^ where slight temperature changes impact performance or outcomes. Traditional contact-based sensors often face limitations when measuring temperature in harsh environments, at micro-to-nanoscale dimensions, or in situations requiring remote monitoring.^5,6^ In this context, luminescent temperature probes based on lanthanide­(III) (Ln^III^) coordination compounds have been extensively studied due to their intense emission and well-established temperature-dependent luminescence behavior.^7–12^ These features enable accurate and high-resolution thermal readouts, making them ideal for advanced sensing applications.^13^

Among the Ln^III^ series, europium­(III) is among the most exploited for developing cutting-edge luminescent materials.^14^ The shortcoming faced by Laporte-forbidden 4f–4f transitions is effectively overcome by coordinating Eu^III^ to organic ligands displaying large molar absorptivity,^15,16^ which enables intramolecular energy transfer (IET) via the well-known antenna effect.^17^ Consequently, Eu^III^ coordination compounds have been investigated as luminescent temperature probes, since their intense emission facilitates the detection of temperature-dependent luminescent outputs.^18–21^ To monitor temperature in these systems, the main protocols rely on employing the intensity ratio of two emission bands (ratiometric) or the ^5^D_0_ level lifetime as the thermometric parameter.^22–24^ Considering the spectroscopic properties of Eu^III^, the most prominent possibility of ratiometric thermometry involves exploiting the emission bands coming from the thermally coupled ^5^D_0_ and ^5^D_1_ levels.^25^ Yet, emissions arising from the ^5^D_1_ level in Eu^III^ complexes are typically weak due to the susceptibility to nonradiative deactivation. Another approach to ratiometric thermometry relies on the intensity ratio between ligand-centered emission bands and Eu^III^ 4f transitions.^26^ However, this process inherently reflects an inefficient ligand-to-Eu^III^ IET, resulting in weak luminescence.

The temperature dependence of the ^5^D_0_ level lifetime, in turn, enables self-calibrated temperature monitoring in Eu^III^ complexes, since lifetime measurements are minimally affected by external conditions.^27,28^ In this case, the thermal response is influenced by the mechanisms responsible for the nonradiative deactivation of the ^5^D_0_ emitting level, which may include multiphonon deactivation, cross-relaxation, ligand-to-metal charge transfer (LMCT) states, and Eu^III^-to-ligand back energy transfer (BET), among others.^29–31^ Therefore, understanding and controlling nonradiative processes to fine-tune the thermometric response of Eu^III^ complexes represents a promising strategy for developing higher-performance probes.

To precisely investigate the influence of nonradiative deactivation on the thermal response of Eu^III^ complexes, it is necessary to establish a well-defined chemical platform that allows meaningful comparisons and facilitates reliable opto-structural correlations. This could be achieved in dinuclear Eu^III^ species with the same bridge and different terminal ligands. As example, Bellucci and co-workers investigated the dinuclear [Eu_2_(β-diketonate)_6_(N-oxide)_*y*_] complexes and demonstrated that the thermal quenching of luminescence is assisted by BET from Eu^III^ to the ligand triplet state and LMCT states.^32^ In another case, Bazi and co-workers demonstrated the role of Pt-centered LMCT states in modulating the temperature sensitivity of the [Eu_2_(tta)_6_(μ-pyrzMOPtClppy)_2_] complex (Htta: thenoyltrifluoroacetone; pyrzMO: pyrazine-N oxide; ppy: 2-phenylpyridine).^33^ The 2,2′-bipyrimidine (bpm) has been used to bridge Ln^III^ ions, since it displays two bidentate coordination pockets that lead to stable complexes.^34,35^ On the other hand, β-diketonate ligands are routinely applied as terminal ligands in these complexes^36–38^ owing to their suitable triplet state energy to enable IET, large molar absorption coefficient, and desirable thermal stability.^39–44^ The 1,3-diphenyl-1,3-propanedionate (dbm^–^) and 4,4,4-trifluoro-1-phenyl-1,3-butanedionate (btfa^–^) ligands are among the most used β-diketones to sensitize Eu^III^ luminescence. Ilmi and co-workers recently reported the use of btfa^–^ to synthesize [Eu_2_(bpm)­(btfa)_6_] and investigated its red electroluminescence,^45^ while Jang and co-workers employed dbm^–^ to prepare the analogous complex [Eu_2_(bpm)­(dbm)_6_].^46^ It is worth stressing that no thermometry study was performed for these systems.

Building on the potential of Eu^III^ complexes as luminescent temperature probes, this work aims to use the terminal ligand in dinuclear complexes to induce different nonradiative contributions from the ^5^D_0_ emitting level and modulate the thermometric response. For this purpose, dbm^–^ or btfa^–^ were employed as terminal ligands to synthesize the complexes [Eu_2_(bpm)­(dbm)_6_] (**1**) and [Eu_2_(bpm)­(btfa)_6_] (**2**). This approach enables mapping the influence of different crystal structure (packing, local symmetry, and internuclear distances), as well as the effect of the ligand backbone on the nonradiative deactivation of the Eu^III^ emitting level. Additionally, it allows assessment of how these factors affect both the thermal quenching range of the luminescence and the relative thermal sensitivity, using the ^5^D_0_ level lifetime as a thermometric parameter.

## Results and Discussion

### Synthesis and Structural Description

The complexes [Eu_2_(bpm)­(dbm)_6_] (**1**) and [Eu_2_(bpm)­(btfa)_6_] (**2**) were synthesized in a one-pot procedure by refluxing the deprotonated β-diketonate ligand, bpm, and EuCl_3_ in ethanol for 2 h. The solution was filtered and allowed to rest at room temperature for 1 week, yielding rectangular yellowish-transparent crystals suitable for single-crystal X-ray diffraction (SC-XRD) analysis. Analogous Gd^III^ complexes were synthesized by following the same procedure. Additional details on synthesis and characterization are provided in Supporting Note S1.

**1** and **2** were obtained as dinuclear structures (Figure 1a,1b); further crystallographic data can be found in Table S1. **1** crystallizes in the monoclinic crystal system (*P*2_1_/*n* space group), while **2** crystallizes in the triclinic system (*P*1̅ space group); no lattice solvent molecules were observed in either structure. The crystal system of **2** matches that previously reported by Ilmi and co-workers,^45^ while **1** crystallizes in the same space group reported by Jang and co-workers,^46^ but lacks diethyl ether molecules in the lattice. One should note that the different crystalline systems of the complexes reflect a larger cell volume for **1** (4122 Å) than **2** (1709 Å); for **1**, the number of formula units per unit cell (Z) is two, while for **2**, Z is one. These results confirm that the β-diketonate terminal ligand influences the crystallization behavior of the dinuclear complexes.

**Figure 1:**
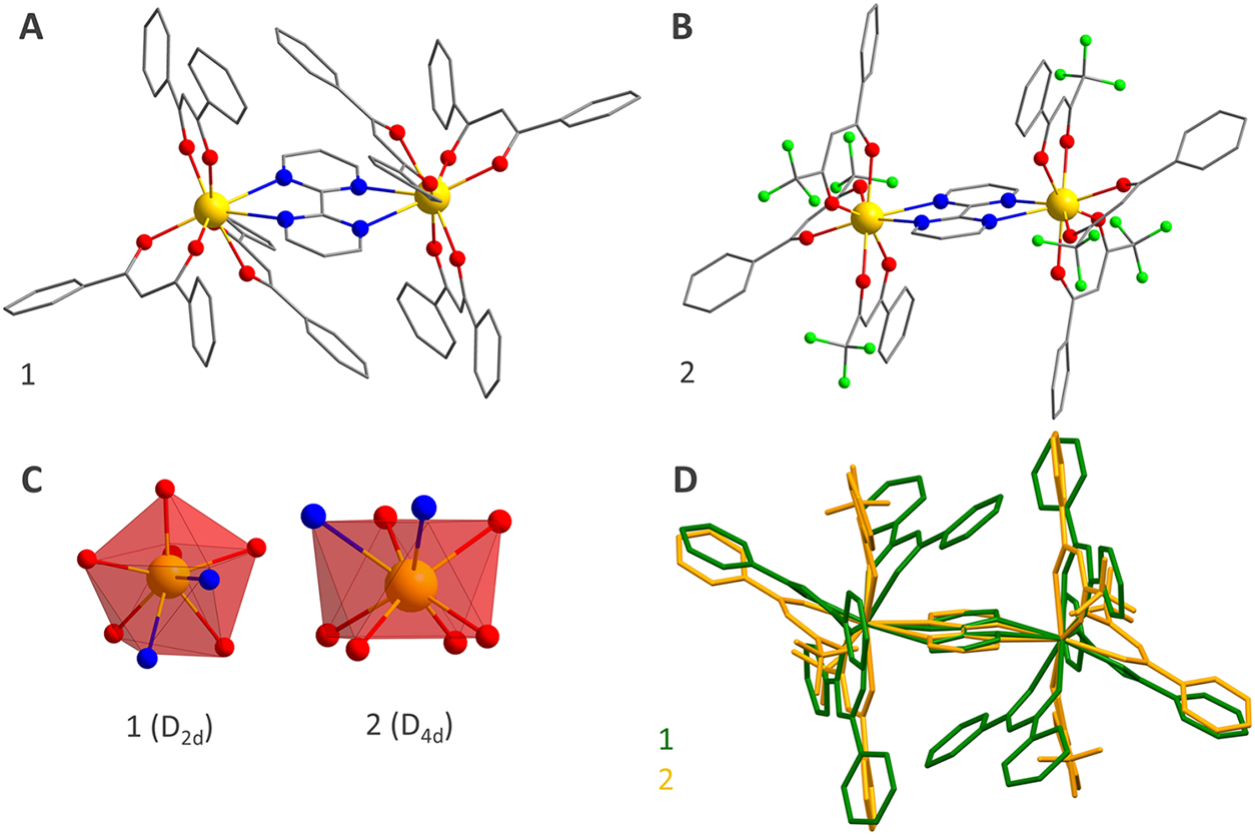
Molecular structure of (A) [Eu_2_(bpm)­(dbm)_6_] (**1**) and (B) [Eu_2_(bpm)­(btfa)_6_] (**2**); hydrogen atoms have been omitted for the sake of clarity. (C) Comparison of the actual Eu^III^ polyhedron to the idealized (in red) triangular dodecahedron polyhedron (TDD-8, D_2d_ point group) for **1** or square antiprismatic (SAP, D_4d_ point group) for **2**. (D) Molecular overlay of **1** and **2**; the fit was performed over the Eu­(bpm)Eu arrange (r.m.s. deviation = 0.0151).

The molecular structure of both complexes is described by the bidentate bpm ligand bridging two symmetry-equivalent Eu^III^ centers, while three β-diketonate ligands fulfill the coordination sphere in the terminal positions (Figure 1a,1b). The bpm ligand coordinates through nitrogen atoms, while the β-diketonate ligands bind via oxygen atoms. This coordination environment leads to a coordination number of eight (EuN_2_O_6_ polyhedron) well described by distorted D_2d_ (triangular dodecahedron) or D_4d_ (square antiprism) local symmetries for **1** or **2** respectively (Figure 1c), according to SHAPE analysis (Table S2).^47^ The Eu–N (from bpm) bond distances are within the 2.6–2.8 Å range, and they are slightly longer for **1** than **2** (Table S3). In contrast, the Eu–O bond lengths are approximately 2.3 Å, noticeably shorter than the Eu–N bond lengths (Table S3). This is not surprising, considering the ionic character of the Eu–ligand bonds and the charge of the β-diketonate moiety compared to the neutral bridge ligand. Moreover, the average Eu–O bond lengths are shorter in **1** than **2** (Table S3) due to the electron-withdrawing effect played by CF_3_ groups in btfa^–^, decreasing the electron density at the coordinated oxygen atoms compared to **1**. Variations in bond distances within the first coordination sphere result in slightly different bite angles for both bpm and β-diketonate ligands (Table S4), contributing to deviations from ideal coordination geometry, as indicated by SHAPE analyses (Table S2). The modifications induced by the ligand scaffold are highlighted by the superimposed molecular structures of both complexes (Figure 1d).

It is now evident that the terminal ligand plays a key role in directing the short-range organization of the molecular structure in dinuclear species. Understanding the origin of this influence is essential for establishing opto-structural correlations aimed at elucidating the thermometric behavior. Accordingly, long-range inter- and intramolecular interactions were thoroughly analyzed to assess how the β-diketonate ligand governs the three-dimensional packing arrangement (Figures S1 and S2). The Eu···Eu intramolecular separations are 6.944 Å for **1** and 6.912 Å for **2** (Table S5), values that fall within the typical range observed for other bpm-bridged {Ln_2_} complexes.^48–50^ However, the intermolecular Eu···Eu distances (Table S5) are significantly shorter in **1** (7.89 Å) than those in **2** (9.32 Å), reflecting the influence of their distinct crystal systems.

The inter- and intramolecular interactions were carefully analyzed to map long-range contacts between the molecules. Although **1** does not display any intramolecular H-bond, **2** exhibits several of these interactions between fluorine atoms in the CF_3_ groups and H atoms of phenyl rings in the btfa^–^ ligand (Figure 2a). These contacts are within the 2.4–3.2 Å range (Table S7), which is typical of moderate-to-weak H-bonds.^51^ Besides the intramolecular interactions, intermolecular H-bonds are detected in both structures. **1** presents O···H interactions between the H atoms of the phenyl ring and oxygens of the dbm^–^ ligand (Figure S3 and Table S6). **2**, in its turn, displays several O···H and F···H intermolecular contacts ranging from 2.5 to 3.2 Å (Figure S4 and Table S7). Apart from the H-bonds, intermolecular F···F interaction (R_1_-F···F-R_2_) takes place between the fluorine atoms of two neighbor btfa^–^ ligands (Figure 2c), whose shortest F···F distance (2.882 Å) and donor–acceptor angles (99.7°) are in good agreement with other systems exhibiting such interaction.^52,53^

**Figure 2:**
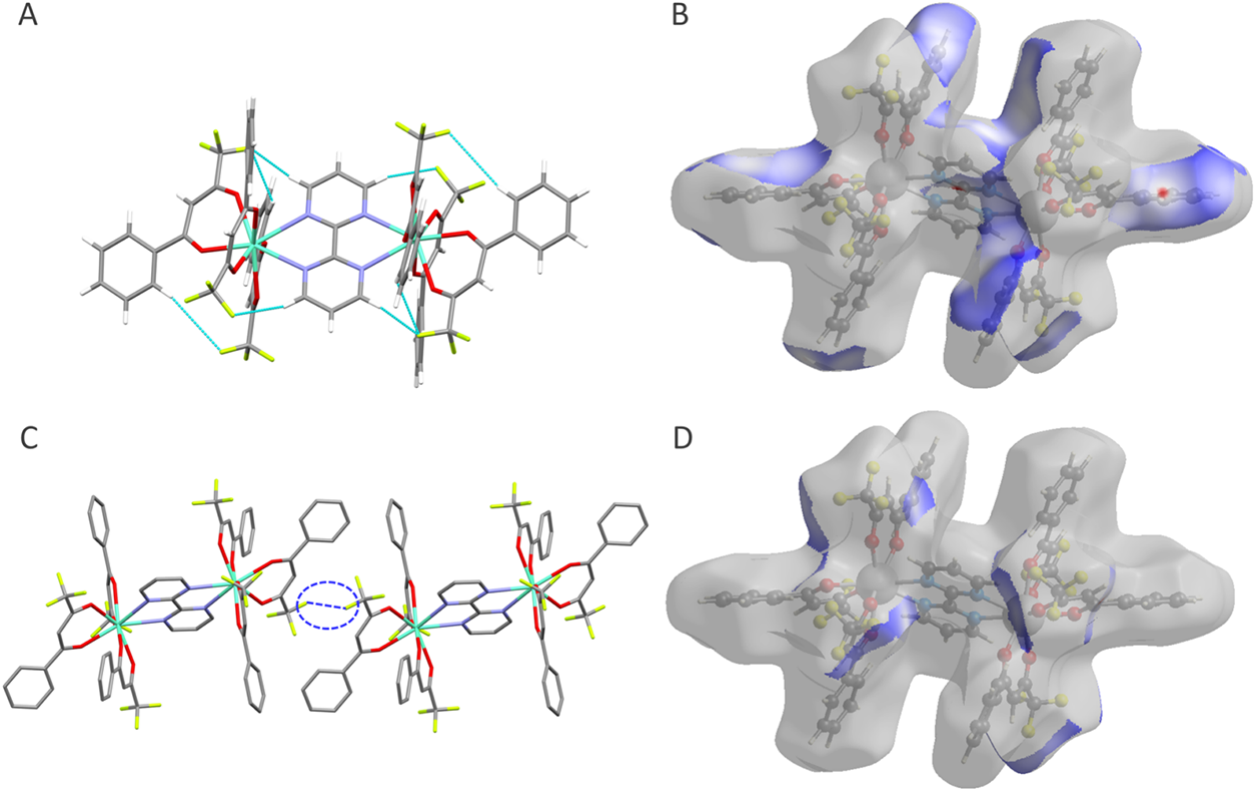
(A) Intramolecular H-bonds (cyan dashed lines) in [Eu_2_(bpm)­(btfa)_6_] (**2**) and (B) Hirshfeld surface highlighting the H···F hydrogen bonds. (C) Intermolecular F···F contacts (blue dashed lines) in **2** and (D) Hirshfeld surface highlighting the F···F contacts in **2**.

Hirshfeld surface analyses performed using CrystalExplorer software^54^ were employed to further investigate the inter- and intramolecular interactions (Figures S5, S6, and 2b,2d). In **1**, the most significant contacts involve the hydrogen and oxygen atoms of the ligand, whereas in **2**, strong interactions also occur in the proximity of the fluorine atoms. The 2D fingerprint plots of all interatomic interactions (Figure S5) show that hydrogen bonds account for only 5.9% of the total surface interactions in **1**, with H···H and C···H contacts constituting the majority. On the other hand, F···H, O···H, and F···F contacts are responsible for 39.1, 6.3, and 6.2% of the overall surface interactions in **2**, respectively (Figures S6 and 2b,d), highlighting the important role of fluorine and H-bond interactions in controlling the crystalline packing.

With a clear understanding of the forces governing molecular organization in the crystals, it is evident that H-bonds and F···F contacts are stronger in **2**, resulting in a more tightly packed structure. This is evidenced by the lower cell volume and larger density of **2** (Table S1). Therefore, the electron-withdrawing effects of the different β-diketonate ligands, along with contrasting inter- and intramolecular interactions, result in distinct Eu–O bond lengths and angles in the two complexes, modifying the local symmetry of the EuN_2_O_6_ coordination polyhedra.

The bulk composition of the complexes was analyzed using both powder X-ray diffraction (PXRD) and Fourier-transform infrared spectroscopy (FTIR). The PXRD pattern recorded for the ground crystals is in good agreement with the simulated pattern derived from SC-XRD data (Figure S7), confirming that the sample retains its crystallinity after grinding. FTIR spectra of the complexes (Figure S8) exhibit characteristic vibrational modes of the C=O bonds around 1590 cm^–1^, attributed to the keto–enol equilibrium of the β-diketones,^55^ as well as vibrations at 1455 and 1458 cm^–1^ related to C–C stretching in the aromatic ring.^56^ A set of bands ranging from weak to strong intensity are observed at 1146 cm^–1^ (**1**) and 1144 cm^–1^ (**2**), 1061 cm^–1^ (**1**) and 1060 cm^–1^ (**2**), 1023 cm^–1^ (**1**) and 1024 cm^–1^ (**2**), and 940 cm^–1^ (**1**) and 942 cm^–1^ (**2**) assigned to deformation of the C–H bond in the aromatic ring.^56^ For **2**, characteristic C–F stretching vibrations from the btfa^–^ ligand are observed in the range from 1381 to 1282 cm^–1^.^45^ Moreover, the bands peaking at 507 and 509 cm^–1^ are assigned to in-plane bending of the C=C bond in the phenyl group. Finally, the bands at 427 cm^–1^ (**1**) and 431 cm^–1^ (**2**) are related to the Eu–O bond, which is consistent with the coordination of the β-diketonate ligands to Eu^III^.^57^ One should note that the FTIR of the Gd^III^ analogous are quite similar to those of the respective Eu^III^ complexes (Figure S8), ensuring the same molecular organization.

For a system to be used as a temperature probe, it must withstand a specific temperature range without degradation. Thermogravimetry measurements (Figure S9) confirm that the two complexes are thermally stable up to 230 °C, enabling temperature-dependent studies within this range. Above this temperature, a thermal degradation takes place, probably associated with the decomposition of the β-diketonate and bpm ligands, in accordance with the behavior displayed by other β-diketonate complexes.^58,59^ Moreover, differential scanning calorimetry (DSC) confirms that no phase transition occurs in the 0–150 °C temperature range (Figure S10); only a minor thermal event is detected, which is likely associated with the release of solvent adsorbed on the crystal surface, since no lattice solvent was identified by SC-XRD. Above 200 °C, an endothermic event at 255 °C (**1**) or 215 °C (**2**) is observed, followed by an exothermic event at 406 °C (**1**) or 326 °C (**2**). These processes are in good agreement with the thermal decomposition of the complexes, as evidenced by thermogravimetric analysis (Figure S9).

The absorption features of the complexes were investigated by diffuse reflectance spectroscopy (DRS, Figure S11), which reveals broad absorption bands within the UV spectral range, assigned to π–π* electronic transitions centered on the β-diketonate and bpm ligands.^45^ The lower energy absorption band assigned to the S_1_ ← S_0_ transition peaks at 355 nm (28,169 cm^–1^) for **1** and at 330 nm (30,303 cm^–1^) for **2**, indicating that the terminal ligand influences the S_1_ state energy of the complexes. Other contributions, including LMCT states, may also influence the absorption features. An effective strategy to assess this effect is to compare the DRS of the Eu^III^ complexes with those of an isostructural Gd^III^ analogue. In the case of Gd^III^, the metal-centered excited states lie at significantly higher energies than the usual ligand triplet levels, thereby minimizing the likelihood of LMCT contributions.^60,61^ Interestingly, the DRS spectra of **1** and **2** are quite similar to those of their Gd^III^ analogues (Figure S11), ensuring that no LMCT states contribute to the absorption behavior of the complexes.

### Photoluminescence and Photophysical Parameters

Prior to evaluation of the influence of the terminal ligand on luminescence thermometry, a detailed investigation of the luminescent properties was carried out to understand the emission dynamics of the complexes. The excitation spectra of **1** and **2** (Figure 3a) display a broad band within the 250–400 nm spectral region, corresponding to S_n_ ← S_0_ absorptions centered in the β-diketonate and bpm ligands.^45^ It is worth noting that the excitation spectra closely resemble the diffuse reflectance spectra (DRS) of the two complexes (Figure S11). In the excitation spectra, additional weaker-intensity bands are observed at 464 and 533 nm, assigned to the ^5^D_2_ ← ^7^F_0_ and ^5^D_1_ ← ^7^F_1_ transitions of Eu^III^, respectively.

**Figure 3:**
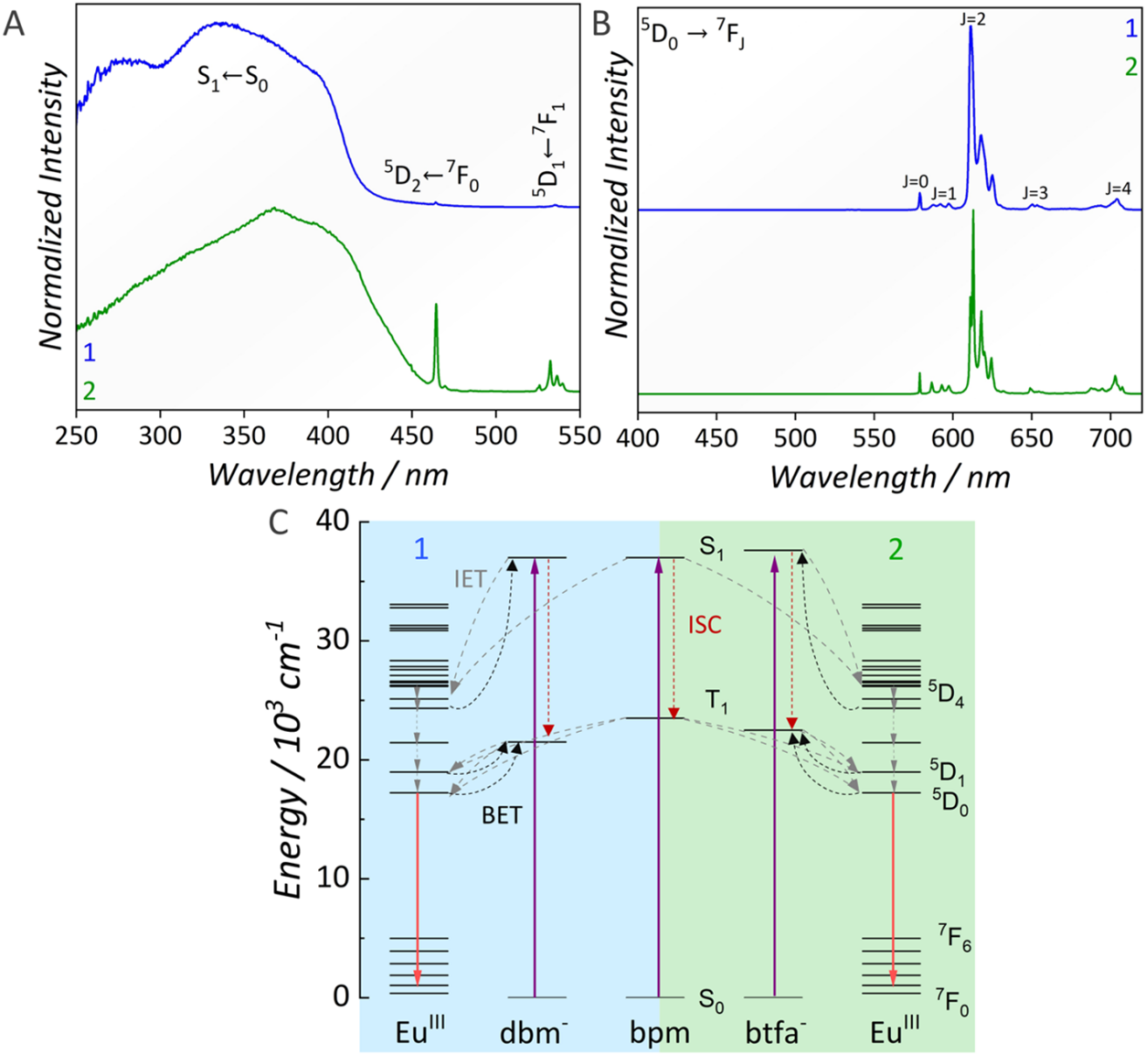
(A) Excitation spectra of [Eu_2_(bpm)­(dbm)_6_] (**1**) (λ_em_ = 612 nm) and [Eu_2_(bpm)­(btfa)_6_] (**2**) (λ_em_ = 612 nm). (B) Emission spectra of **1** (λ_ex_ = 330 nm) and **2** (λ_ex_ = 370 nm). All spectra were collected for crashed crystals at 300 K. (C) Partial energy diagram illustrating the ligand-centered singlet (S_1_) and triplet (T_1_) levels, alongside the ^2S+1^L_J_ levels of Eu^III^. The diagram highlights ligand-centered S_n_ absorption (purple arrows), intersystem crossing (ISC, red dashed arrows), ligand-to-Eu^III^ intramolecular energy transfer (IET, gray dashed arrows), Eu^III^-to-ligand back energy transfer (BET, black dashed arrows), and Eu^III^ emission (red arrows).

The emission spectra of **1** and **2** collected at 300 K (Figure 3b) exhibit narrow emission bands in the 570–720 nm spectral region, assigned to a set of Eu^III 5^D_0_ → ^7^F_J_ (*J* = 0–4) transitions. The most intense emission, attributed to the ^5^D_0_ → ^7^F_2_ transition, gives rise to the characteristic red luminescence of Eu^III^ under UV excitation (Figure S12). For both complexes, no ligand-centered emission bands are observed around 400 nm, indicating that the β-diketonate ligands effectively sensitize Eu^III^ luminescence via an antenna effect. This is not surprising considering that both dbm^–^ and btfa^–^ ligands are well-known sensitizers of Eu^III^, since their typical triplet state energy (T_1_) are similar and close enough to the excited levels of Eu^III^ to enable IET;^62,63^ the bpm ligand also presents adequate T_1_ energy for sensitizing the Eu^III^ luminescence.^64^ Ilmi and co-workers previously reported the T_1_ energy of [Eu_2_(bpm)­(btfa)_6_] as 22,531 cm^–1^ ^45^ while the triplet state energy of dbm^–^ is expected to be close to 21,600 cm^–1^.^65^

Ilmi and co-workers^45^ also investigated the ET mechanisms in **2**, concluding that the process is predominantly governed by IET from triplet states mainly localized on the btfa^–^ ligand, as well as from mixed triplet states involving both btfa^–^ and bpm ligands. IET between Eu^III^ centers within the structure is also expected to occur, but its contribution is significantly lower than that of the ligand-to-Eu^III^ IET process. BET from Eu^III^ to ligand states occurs mainly from the ^5^D_1_ and ^5^D_0_ states of Eu^III^ to the ligand triple state, as well as from the ^5^D_4_ level to the S_1_ state.^45^ The energy diagram represented in Figure 3c summarizes the antenna effect played by the ligands, intramolecular energy transfer, back energy transfer, and the Eu^III^ emission. In this diagram, ligand–centered absorption at 250–400 nm populates the singlet (S_n_) states of the ligands, followed by intersystem crossing to T_1_. Then, ligand-to-Eu^III^ IET occurs (with a possible S_1_ contribution), followed by Eu^III^ emission from the ^5^D_0_ level.

Further insights into the luminescence behavior of the complexes were obtained from the analysis of their photophysical parameters (Table 1), including the ^5^D_0_ level lifetime (τ), radiative (*A*_rad_) and nonradiative (*A*_nrad_) decay rates, Judd-Ofelt intensity parameters (Ω_2_ and Ω_4_), intrinsic emission quantum yield (Φ_Eu_^Eu^), and absolute emission quantum yield (Φ_Eu_^L^). *Q*_Eu_^Eu^ is the ratio of *A*_rad_ to *A*_total_ (*A*_rad_ + *A*_nrad_), while *Q*_Eu_^L^ stands for the ratio of emitted-to-absorbed photons. The full description of the calculations is presented in Supporting Note S2. The Ω_2_ parameter is more sensitive to changes in the local symmetry of Eu^III^ while Ω_4_ is strongly dependent on the covalency of the Eu^III^–ligand bond.^66^ The lower Ω_2_ value for **2** compared to that displayed by **1** suggests that Eu^III^ is inserted in a more symmetrical environment, which agrees with the distorted D_4d_ local symmetry of the EuN_2_O_6_ polyhedra determined from SC-XRD for **2** (Figure 1c). Moreover, the Ω_4_ value is larger for **2**, suggesting a higher degree of covalency in the Eu–ligand bonds compared to **1**.

**Table 1:** ^5^D_0_ Level Lifetime (τ), Judd-Ofelt Intensity Parameters (Ω_2_ and Ω_4_), Radiative (*A*_rad_) and Nonradiative (*A*_nrad_) Decay Rates, Absolute Emission Quantum Yield (Φ_Eu_^L^), Intrinsic Emission Quantum Yield (Φ_Eu_^Eu^), and Sensitization Efficiency (η = Φ_Eu_^L^/Φ_Eu_^Eu^) Determined for [Eu_2_(bpm)­(dbm)_6_] (**1**) and [Eu_2_(bpm)­(btfa)_6_] (**2**) as Crystals

complex	Ω_2_/10^–20^ cm^2^	Ω_4_/10^–20^ cm^2^	τ/ms	*A*_rad_/s^–1^	*A*_nrad_/s^–1^	Φ_Eu_^Eu^/%	Φ_Eu_^L^/%	η/%
**1**	30	5.9	0.435	1091	1204	47.5	0.5	1.05
**2**	13	8.1	0.753	602.8	725.2	45.3	27.7	61.0

The luminescence kinetics of the complexes at 300 K was investigated by time-resolved spectroscopy (Figure S13) to determine the ^5^D_0_ level lifetime. The emission decay curves were undertaken by monitoring the ligand excitation and the Eu^III 5^D_0_ → ^7^F_2_ emission transition. The emission decay curves of both complexes were fitted with a monoexponential function, yielding decay time constants of τ = 0.435 ms (**1**) and τ = 0.753 ms (**2**) (Table 1). The lifetime is a kinetic parameter related to the radiative and nonradiative decay rates from the ^5^D_0_ emitting level, according to the relation τ = (*A*_rad_ + *A*_nrad_)^−1^. The different lifetimes observed in the two complexes indicate that the deactivation process at the Eu^III 5^D_0_ level is strongly influenced by the terminal ligand. The *A*_rad_ for **1** is slightly larger than that of **2** (Table 1); yet its nonradiative decay rate is considerably larger as evidenced by the shorter lifetime (Table 1). One should note that the more rigid structure displayed by **2** due to stronger H-bonds and F···F contacts decreases de contribution of vibrational quenching, thereby accounting for the lower *A*_nrad_ of **2**.^30^ Moreover, the different energy of the S_1_/T_1_ state also influences the BET, thus, changing *A*_nrad_.

The intrinsic emission quantum yield (Φ_Eu_^Eu^) of both complexes is close to 45% (Table 1). However, the absolute emission quantum yield (Φ_Eu_^L^) of **1** is lower than 1% (Table 1), in agreement of other dbm-based Eu^III^ complexes.^67^ On the other hand, Φ_Eu_^L^ for **2** is 27.7%, corroborating the values reported for similar Eu^III^ complexes.^68,69^ Furthermore, the sensitization efficiency (η = *Q*_Eu_^L^/*Q*_Eu_^Eu^)^70^ of **2** is considerably larger than in **1** (Table 1), due to the lower contribution of nonradiative pathways.

To further investigate the photoluminescence dynamics of the complexes at 300 K, a molecular dilution was employed to try to disentangle the effects of the inter- and intramolecular interactions. For that, the photoluminescence features were recorded for the complexes dissolved in chloroform (1 mg mL^–1^) or dispersed in poly­(methyl methacrylate) (PMMA) films. The excitation and emission spectra are shown in Figure S14 while the emission decay curves are presented in Figure S13. The excitation and emission spectra of the complex in the PMMA films are quite similar to those in the crystals; however, in solution, the excitation bands shift toward longer wavelengths. This effect can be assigned to a preferential stabilization of the excited states over the ground level due to interaction of the ligands with solvent molecules. In addition, subtle conformational changes of the complex are expected to modify the ligand singlet and triplet state energies, thereby altering the position of the excitation bands.^71,72^

The emission decay curves of the complexes in chloroform or PMMA (Figure S13) were fitted by a biexponential function, indicating the presence of more than one nonequivalent deactivation process from the Eu^III 5^D_0_ emitting level. This behavior may be associated with the existence of different conformers of the complex. Indeed, we have previously discussed that Eu^III^ β-diketonate complexes dispersed in PMMA tend to assume more than one conformation due to the interaction with the polymeric chains.^73^ The two ^5^D_0_ level lifetime components for each system were used to calculate an average lifetime according to eq S5. The ^5^D_0_ level lifetimes (Table S8) are shorter in both solution and PMMA films than in the crystals, ensuring that the radiative and nonradiative balance is altered upon dilution. After dilution, the change of the *A*_rad_ value (Table S8) can be assigned to the different refractive index of the medium (complex = 1.5; chloroform = 1.445;^74^ PMMA = 1.490)^75^ since this parameter influences the radiative decay rate according to eq S1; slight changes in the conformation of the complexes are also expected to modify the Eu^III^ first coordination sphere, and thus, the radiative rate.

The *A*_nrad_ values increase after dilution of the complexes in both chloroform or PMMA (Table S8). For the PMMA films, we recently discussed that multiphonon deactivation induced by the PMMA chains also influences the nonradiative deactivation of the ^5^D_0_ emitting level.^73^ In solution, on the other hand, the stabilization of singlet/triplet states could potentially change the ligand-to-Eu^III^ IET dynamics, thus modifying the deactivation pathways of the ^5^D_0_ emitting level.^71,72^ One should note that the ^5^D_0_ lifetime of **1** in chloroform is strongly suppressed by vibrational quenching, abruptly increasing the *A*_nrad_ (Table S8). This effect is less pronounced in **2**, as this complex can establish a greater number of hydrogen bonds with the solvent, thereby increasing its structural rigidity. Therefore, although both PMMA and chloroform influence the luminescence dynamics of the complexes, dilution in chloroform suggests that **2** has a greater tendency to present stronger H-bonds, which increase its rigidity.

It is now clear that the terminal ligand influences the photophysical dynamics of bpm-bridged dinuclear Eu^III^ complexes. Among the two terminal ligands employed in this study, dbm^–^ enhances the nonradiative contribution from the ^5^D_0_ emitting level. This effect could be rationalized based on the ligand scaffold combined with insights from the crystal and electronic structure. The replacement of one phenyl ring containing C–H bonds in dbm^–^ by C–F bonds in btfa^–^, which display lower vibration energy, decreases the probability of multiphonon processes through C–H oscillators that quench the Eu^III^ emission from the ^5^D_0_ level.^76^ Furthermore, the more rigid structure displayed by **2** diminishes the contribution of vibrational quenching, decreasing *A*_nrad_ compared to **1**. Changes in the S_1_ and T_1_ energies induced by the ligands also alter the IET and BET dynamics. These changes are expected to influence the thermal behavior of luminescence and, consequently, the thermometric properties, as discussed below.

### Luminescence Thermometry

To test our hypothesis that thermometric behavior can be tuned by modulating nonradiative processes via the terminal ligand in dinuclear Eu^III^ complexes, we investigated the temperature dependence of luminescence for both systems. For that, the temperature dependence of the ^5^D_0_ level lifetime was monitored to provide further insight into the deactivation mechanisms. Emission decay curves (Figure 4) were recorded from 77 to 420 K for **1** and from 77 to 440 K for **2** and they were fitted by a monoexponential function to calculate the ^5^D_0_ lifetime (Figure 5a,5b). The ^5^D_0_ lifetime remains nearly constant up to 260 K (**1**) or 300 K (**2**), after which it becomes shorter. This comparison shows that thermally induced quenching of luminescence occurs at lower temperatures in **1**, reflecting a more significant temperature-driven nonradiative deactivation of the ^5^D_0_ excited level than in **2**.

**Figure 4:**
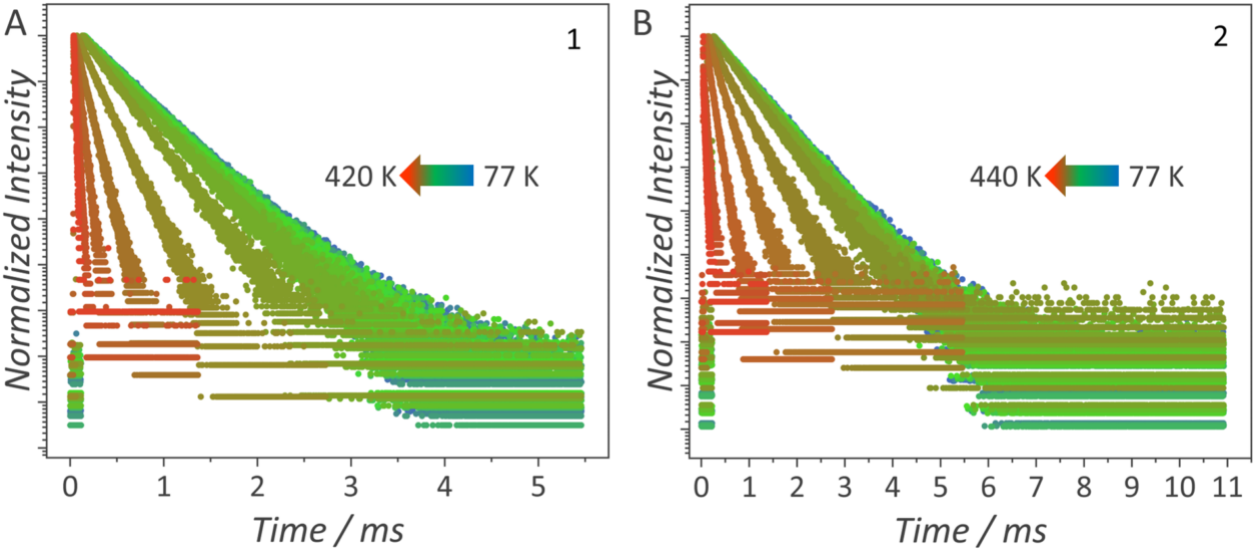
Temperature-dependent emission decay curves of (A) [Eu_2_(bpm)­(dbm)_6_] (**1**) monitored at λ_exc_ = 330 nm, λ_em_ = 612 nm and (B) [Eu_2_(bpm)­(btfa)_6_] (**2**) monitored at λ_exc_ = 370 nm, λ_em_ = 612 nm. The data were obtained for the complexes as crystals.

**Figure 5:**
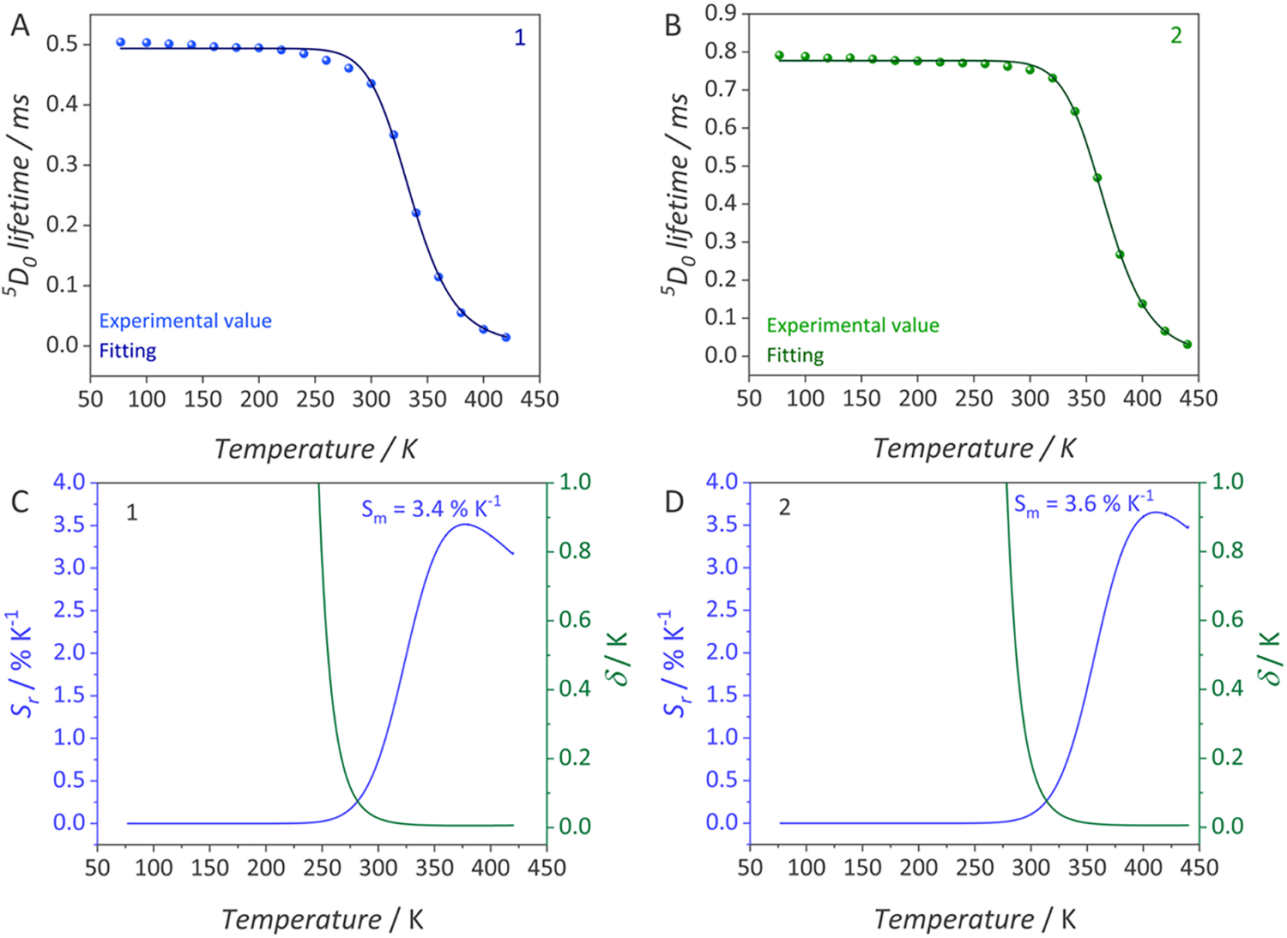
Temperature dependence of the ^5^D_0_ level lifetime (τ) used as thermometric parameter (Δ) and best fits to the Mott-Seitz function for (A) [Eu_2_(bpm)­(dbm)_6_] (**1**) and (B) [Eu_2_(bpm)­(dbm)_6_] (**2**) as crystals; fitting parameters are listed in Table 2. Relative thermal sensitivity (S_r_) and temperature uncertainty (δ*T*) for (C) **1** and (D) **2**. For the sake of clarity, the δ*T* values are displayed only when they are below 1 K.

As the temperature increases, luminescence tends to diminish due to multiple quenching pathways. These may involve BET from Eu^III^ to the ligands^77^ or LMCT states,^20^ for instance. For both complexes, the diffuse reflectance spectra (Figure S11) show no evidence of LMCT states; therefore, this pathway can be ruled out. Consequently, BET from the Ln^III^ center to the ligands should be the main pathway for temperature quenching, as reported for other dinuclear Eu^III^ complexes.^32^

The temperature dependence of the ^5^D_0_ lifetime typically follows the well-known Mott-Seitz model. In this mechanism, part of the population in the excited level decays nonradiatively to the ground level through the intersection of the potential energy curve of the excited level with that of the ground level (or intermediated by other excited levels), driven by thermal energy.^20,78,79^ The temperature-dependent ^5^D_0_ lifetime curves were fitted by a single Mott-Seitz function (eq 1). In eq 1, τ_0_ represents the intrinsic radiative lifetime (extrapolated to 0 K), α denotes the *A*_nrad_/*A*_rad_ ratio, *K*_B_ is the Boltzmann constant, and ΔE corresponds to the activation energy associated with thermal quenching (Table 2).

1 $$\tau =\frac{\tau_{0}}{1+\alpha \exp\left(-\frac{\Delta E}{K_{B}T}\right)}$$

**Table 2:** Fitting Parameters Obtained from the Mott-Seitz Function (eq 1), Describing the Temperature Dependence of the ^5^D_0_ Lifetime for [Eu_2_(bpm)­(dbm)_6_] (**1**) and [Eu_2_(bpm)­(btfa)_6_] (**2**) as Crystals or PMMA Films

			PMMA	
complex	parameter	crystal	process 1	process 2
**1**	τ_0_/s	0.493 ± 0.002	0.616 ± 0.009	0.401 ± 0.006
	α/s^–1^	2.79 ± 0.2 × 10^7^	2.58 ± 0.1 × 10^6^	6.9 ± 0.1 104
	Δ*E*_1_/cm^–1^ (meV)	3999 ± 212 (495)	3312 ± 481 (410)	2268 ± 252 (281)
**2**	τ_0_/s	0.777 ± 0.02	0.730 ± 0.02	0.389 ± 0.007
	α/s^–1^	1.84 ± 0.1 × 10^8^	8.86 ± 0.1 × 10^5^	2.6 ± 0.1 × 10^6^
	Δ*E*/cm^–1^ (meV)	4963 ± 175 (615)	3348 ± 403 (414)	3531 ± 584 (437)

Fitting the data of **1** using the Mott–Seitz model reveals that the activation energy of the thermal quenching determined for **2** (4963 ± 175 cm^–1^) is larger than that obtained for **1** (3999 ± 212 cm^–1^), indicating a greater thermal barrier for luminescence quenching in **2**. This finding accounts for the higher temperature range at which luminescence quenching occurs in **2** and aligns with the stronger nonradiative deactivation rate previously observed for **1** (Table 1). Considering the contribution of BET to the deactivation of the ^5^D_0_ emitting level, and the absence of LMCT states, it is feasible to assume that the BET pathway is the main contribution to the thermal quenching of luminescence in both complexes, as reported for other Ln^III^ complexes.^77^ Variations in the activation energies between the two complexes arise from subtle differences in the S_1_ and T_1_ states of the ligands, which modulate the energy gap between the ligand states and the Eu^III^ excited levels. One should note that the *A*_nrad_/*A*_rad_ ratio (α) also plays a role in the lifetime value, as described by eq 1. Thus, these findings demonstrate that modifying the terminal ligand in dinuclear complexes enables control over the temperature range of thermal quenching by modulating the nonradiative deactivation of the ^5^D_0_ excited level and the activation energy of the quenching process.

The thermal dependency of luminescence was also studied for the complexes dispersed in the PMMA films, as represented in Figure S15. As expected, two distinct lifetime values were determined, in agreement with the trend observed at 300 K. The thermal dependency of the ^5^D_0_ lifetimes for each complex was fitted by following the Mott–Seitz model (Figure S16), rendering lower activation energy to the thermal quenching processes than the free-standing crystals (Table 2). This observation agrees with the higher nonradiative deactivation rate determined for the complexes in the PMMA films at 300 K (Table S8). The decrease of the activation energy is likely associated with the enhanced multiphonon deactivation induced by the interaction with the PMMA chains and changes of the singlet/triplet state energies, as previously reported by us for similar Eu^III^ β-diketonate complexes.^73^

The ^5^D_0_ lifetime was employed as the thermometric parameter (Δ) to assess the potential of the complexes as luminescent thermal probes (Figure 5a,b). The Mott–Seitz model was applied to calculate the relative thermal sensitivity (S_r_) and the temperature uncertainty (δ*T*), as depicted in Figure 5c,d; further details of the calculation are provided in Supporting Note S2. For **1**, the maximum relative thermal sensitivity (*S*_m_) reached 3.4% K^–1^ at 370 K, whereas for **2**, *S*_m_ was 3.6% K^–1^ at 410 K, with minimum uncertainties of 0.02 K for both complexes. The *S*_m_ values are comparable to those of most Eu^III^-based luminescent temperature probes reported in Table 3. Moreover, the low δ*T* values are expected, given that a highly sensitive PMT detector was employed in the measurements.^80^ Interestingly, the operational temperature range of the complexes as temperature probes can be tuned by the terminal ligand through its influence on the nonradiative thermal quenching of luminescence: **1** operates from 270 to 420 K, whereas **2** functions from 300 to 440 K.

**Table 3:** State-of-the-Art Luminescent Temperature Probes Based on Eu^III^ Coordination Compounds^a^,^b^

complex	working temperature	*S*_m_/% K^–1^	optical parameter	references
[Eu_2_(bpm)(dbm)_6_]	270–420	3.4	lifetime	this work
[Eu_2_(bpm)(btfa)_6_]	300–440	3.6	lifetime	this work
[Eu(hfa)_3_BDPC]_n_	300–500	2.70	lifetime	^81^
[Eu_0.5_Gd_0.5_(hfa)_3_BDPC]_n_	300–500	3.73	lifetime	^74^
[Eu_0.25_Gd_0.75_(hfa)3BDPC]_n_	300–500	2.97	lifetime	^74^
[Eu(tta)_3_(pyphen)]	298–348	1.7	lifetime	^82^
[Eu(hfa)_3_(dpco)_2_]	300–500	0.62	lifetime	^83^
[Eu(bzac)_3_(H_2_O)_2_]	75–300	1.35	lifetime	^84^
[L_1_Eu]^−^	298–323	1.8	lifetime	^85^
[Eu(dbm)_3_(phen)/PS]	308–333	4.2	ratiometric	^86^
CAB1.5:Eu2	298–393	4.22	ratiometric	^87^
EuT	296–363	5.0	ratiometric	^88^
[(Eu(CPDk_3–5_)_3_phen)]	298–363	27.7	single-intensity	^89^
PHTF:Eu	100–420	4	excitation	^21^

a The maximum relative thermal sensitivity (*S*_m_) is used as a figure of merit.

b bpm: 2,2′-bipyrimidine; dbm: 1,3-diphenyl-1,3-propanedionate; btfa: 4,4,4-trifluoro-1-phenyl-1,3-butanedionate; hfa: hexafluoroacetylacetonate; BDPC: 6,12-bis­(diphenylphosphoryl) chrysene; dpco: diphenylphosphoryl chrysene; bzac: 1-phenyl-1,3-butanedione; L_1_: DO3A ligand; phen: 1,10-phenanthroline; pyphen: pyrazino­[2,3-*f*]­[1,10]­phenanthroline; tta: thenoyltrifluoroacetone; PS: polystyrene; CAB: cellulose acetate butyrate, Eu2 = [Eu­(tta)_3_] - tta: thenoyltrifluoroacetone; EuT: [Eu­(tta)_3_(H_2_O)_2_]; CPDk_3–5_: 1-[4-(4-propylcyclohexyl)­phenyl]-octane-1,3-dione; PHTFE: hydrogen-bonded triazine framework.

Overall, the results confirm that the choice of different terminal ligands alters the local coordination environment, crystal packing, and electronic structure of the complexes, thereby influencing the nonradiative deactivation rates of the ^5^D_0_ emitting level and the luminescence thermometry capability. Specifically, dbm^–^ contains more C–H bonds than btfa^–^, enhancing the probability of multiphonon relaxation that quenches the Eu^III^ emission from the ^5^D_0_ level. On the other hand, due to stronger H-bonds and F···F contacts, btfa^–^ induces more tightly packed structure in **2**, decreasing the nonradiative deactivation as well from the Eu^III 5^D_0_ emitting level. The different S_1_ and T_1_ state energies induced by the terminal ligands also influence the intermolecular energy transfer from the ligands to Eu^III^ as well as the back energy transfer processes. The combined structural and electronic effects result in a higher nonradiative rate and a lower activation energy for thermal quenching process in **1**. As a consequence, the operational temperature range of these complexes as luminescent thermometers can be tuned via the terminal ligand: **1** works effectively from 270 to 420 K, whereas **2** operates from 300 to 440 K. This shift directly impacts the practical thermometric operating window, since the usable temperature range is defined not only by the magnitude of *S*_m_ but also by the temperature at which quenching becomes significant and limits emission intensity and measurement reliability.

## Conclusions

This study demonstrates the role of terminal β-diketonate ligands in modulating the nonradiative deactivation and thermal response of luminescence in dinuclear Eu^III^ complexes. By employing dbm^–^ or btfa^–^ as terminal ligands in [Eu_2_(bpm)­(dbm)_6_] (**1**) and [Eu_2_(bpm)­(btfa)_6_] (**2**), respectively, we show that ligand-induced variations in crystal packing, molecular geometry, and electronic structure directly influence the nonradiative deactivation dynamics of the ^5^D_0_ excited level. **1** exhibits a greater number of C–H oscillators, which should increase the probabilities of multiphonon relaxation. **2**, in its turn, displays a more tightly packed structure owing to stronger H-bonds and F···F contacts induced by the btfa^–^ ligand. Slightly different S_1_ and T_1_ state energies of the ligands influence the intermolecular energy transfer from the ligands to Eu^III^ as well as the back energy transfer processes. Consequently, **1** presents higher contribution of nonradiative processes and undergoes thermal quenching of luminescence at lower temperatures compared to **2**. In contrast, the btfa^–^ ligand in **2** decreases the nonradiative deactivation and raises the activation energy for thermal quenching, thereby extending its thermometric range to higher temperatures. These differences result in **1** functioning as a temperature probe from 270 to 420 K (*S*_m_ of 3.4% K^–1^ at 370 K), while **2** operates from 300 to 440 K (*S*_m_ of 3.6% K^–1^ at 410 K). Therefore, the ability to tailor thermometric response via ligand effects on nonradiative deactivation opens new avenues for developing lanthanide­(III)-based optical temperature sensors with customizable working ranges.

## Supplementary Material







## Notes

Synthetic procedure and characterization apparatus, additional structural data, thermogravimetry, diffuse reflectance spectroscopy, and further steady-state and time-resolved photoluminescence spectroscopy data (PDF)
Crystallographic data of compounds (CIF)
Crystallographic data of compounds (CIF)
