# Supplementary material for: Engineering the Thermometric Response of Dinuclear EuIII Complexes via Terminal Ligand-Induced Nonradiative Processes
Source: ACS Omega. 2026 Apr 16;11(16):24449–60. doi: 10.1021/acsomega.6c00433 (PMC13129865; doi:10.1021/acsomega.6c00433)
Supplement: Supplementary file 1 [file ao6c00433_si_001.pdf]

Supplementary material

Engineering the Thermometric Response of Dinuclear Eu^{III} Complexes via Terminal Ligand-Induced Non-radiative Processes

Ariane C. F. Beltrame,[a,b,c] Rodolpho A. N. Silva,[c] Sergio A. M. Lima,[a,b] Luciano Marchiò,[d] Matteo Melegari,[d] Flavia Artizzu,[c] Airton G. Bispo-Jr,[e]* Ana M. Pires [a,b]*

[a] São Paulo State University (Unesp), School of Technology and Sciences, Presidente Prudente, SP 19060-900, Brazil.

[b] São Paulo State University (Unesp), Institute of Biosciences, Humanities and Exact Sciences, São José do Rio Preto SP, 15054-000, Brazil.

[c] University of Piemonte Orientale "Amedeo Avogadro", Department of Sustainable Development and Ecological Transition, Vercelli, VC 13100, Italy.

[d] Department of Chemistry, Life Sciences and Environmental Sustainability, University of Parma, Parco Area delle Scienze 17/A, 43124 Parma, Italy.

[e] University of São Paulo (USP), Institute of Chemistry, Lineu Prestes Street, 748, São Paulo 05508-900, Brazil.

Index

Supplementary note S1 – Experimental procedure 2

Supplementary note S2 – *Data analysis* 5

Supplementary note S3 – Crystallographic data 6

Supplementary note S4 – Thermogravimetry 14

Supplementary note S5 – Additional spectroscopic data 15

Supplementary references 19

Supplementary note S1 – Experimental procedure

Preparation of dinuclear Eu^{III} complex $[Eu_2(bpm)(dbm)_6]$ (1) and $[Eu_2(bpm)(btfa)_6]$ (2)

Both complexes were prepared according to a method reported in literature[1]. For this, the ligand 1,3-diphenyl-1,3-propanedionate (dbm) or 4,4,4-trifluoro-1-phenyl-1,3-butanedionate (btfa) (0.6 mmol) were solubilized in 10 mL of ethanol. Then, a solution of potassium hydroxide 1.1 mol L^{-1} (590 µL, 0.6 mmol) was added to deprotonate the ligand, followed by the addition of the bridging ligand 2,2'-bipyrimidine (bpm) (0.1 mmol). After fifteen minutes, an ethanolic solution of $EuCl_3$ (0.2 mmol) was slowly mixed. The resulting mixture was stirred for 2 h at 60 °C, filtered and allowed to evaporate. A few weeks later, single crystals suitable for single-crystal X-ray diffraction were obtained. Analogous Gd^{III} complexes were synthesized following the same procedure.

$[Eu_2(bpm)(dbm)_6]$ (1): FTIR (ATR cm^{-1}): 416 (w), 427 (m), 454 (w), 507 (s), 535 (w), 604 (w), 607 (s), 618 (w), 654 (m), 683 (s), 698 (w), 711 (s), 715 (w), 735 (m), 758 (w), 769 (w), 784 (m), 814 (w), 843 (w), 851 (w), 928 (w), 940 (m), 973 (w), 987 (w), 1000 (w), 1013 (w), 1023 (s), 1061 (s), 1100 (w), 1028 (w), 1146 (w), 1156 (m), 1177 (m), 1216 (s), 1226 (w), 1246 (w), 1271 (w), 1282 (m), 1309 (m), 1374 (m), 1403 (s), 1442 (w), 1455 (s), 1476 (s), 1509 (s), 1522 (w), 1543 (s), 1561 (w), 1571 (w), 1591 (s), 1619 (w). Anal. Calc. for **1**: C, 65.34; H, 4.03; N, 3.11; %. Found: C, 61.34; H, 4.03, N, 2.35.

$[Eu_2(bpm)(btfa)_6]$ (2): FTIR (ATR cm^{-1}): 431 (w), 463 (m), 509 (m), 579 (s), 630 (s), 661 (m), 677 (w), 681 (w), 693 (s), 716 (m), 754 (w), 759 (s), 765 (w), 773 (w), 796 (w), 809 (w), 813 (w), 833 (w), 841 (w), 924 (w), 942 (s), 968 (w), 985 (w), 1002 (w), 1018 (m), 1024 (m), 1060 (m), 1093 (w), 1127 (s), 1144 (w), 1160 (w), 1181 (s), 1217 (w), 1248 (m), 1282 (s), 1307 (w), 1317 (m), 1381 (w), 1409 (s), 1426 (w), 1439 (w), 1458 (w), 1469 (m), 1489 (m), 1530 (s), 1537 (w), 1573 (s), 1598 (s), 1607 (s), 1627 (w). Anal. Calc. for **2**: C, 46.59; H, 2.42; N, 3.20; %. Found: C, 44.32; H, 2.39, N, 2.97.

$[Gd_2(bpm)(dbm)_6]$: 423 (w), 434 (w), 445 (m), 504 (s), 604(s), 614 (w), 657 (m), 683 (s), 714 (m), 742 (s), 752 (w), 779 (m), 813 (w), 823 (w), 834 (w), 845 (w), 924 (w), 938 (m), 966 (w), 986 (w), 999 (w), 1021 (s), 1064 (s), 1095 (w), 1128 (w), 1146 (w), 1157 (m), 1178 (m), 1217 (s), 1253 (w), 1279 (w), 1297 (m), 1308 (m), 1372 (m), 1381 (m), 1401 (s), 1441 (w), 1455 (s), 1476 (s), 1514 (s), 1542 (s), 1574 (w), 1593 (s), 1620 (w).

$[Gd_2(bpm)(btfa)_6]$: 432 (w), 464 (m), 511 (m), 578 (s), 630 (s), 661 (m), 679 (m), 696 (s), 717 (m), 759 (s), 765 (w), 795 (w), 813 (w), 835 (w), 922 (w), 941 (s), 968 (w), 986 (w), 999 (w), 1024 (m),1060 (w), 1074 (m), 1094 (w), 1129 (s), 1144 (w), 1160 (w), 1180 (s), 1246 (m), 1284 (s), 1307 (w), 1318 (m), 1377 (w), 1410 (s), 1436 (w), 1458 (w), 1471 (m), 1488 (m), 1529 (s), 1538 (w), 1573 (s), 1598 (s), 1607 (s), 1632 (w).

Fabrication of the PMMA films

PMMA films doped with the complexes $[Eu_2(bpm)(dbm)_6]$ and $[Eu_2(bpm)(btfa)_6]$ were prepared according to the procedure described in the literature.[2] Both films were obtained with a

concentration of 1% (m/m) of the complex relative to the PMMA. Initially, solutions of the complexes were prepared in chloroform at a concentration of 1.0 mg mL^{-1}. Then, 1.5 mL of the solution of complex was mixed with 148.5 mg of PMMA previously dissolved in 2.0 mL of chloroform. The resulting mixture was kept under magnetic stirring with heating to partially reduce the solvent volume and obtain a homogeneous solution. The films were then deposited onto quartz substrates (20 × 20 mm^2) and dried in a chloroform-saturated atmosphere to prevent the formation of opaque films.

Characterization

Single-crystal X-Ray diffraction (SC-XRD). SC-XRD was collected at 200 K with a Bruker (US) D8 Venture diffractometer equipped with a Photon III detector, using a microfocus radiation source (Mo Kα: λ = 0.71073 Å). The intensity data were integrated from several series of exposure frames covering the sphere of reciprocal space. Data reductions were performed with APEX5. Absorption corrections were applied using the program SADABS.[3] The structures were solved by intrinsic phasing with the program SHELXT.[4] Fourier analysis and refinement were performed by the full–matrix least–squares methods based on F^2 using SHELXL–2017 implemented in Olex2 software (version 1.5).[5] Non–H atoms were refined anisotropically. H atoms were placed in their calculated positions except for the ones bonded to oxygen in OH, that were found and refined from their electron density on the residual electron density map. [Eu_2(bpm)$(dbm)_6$] (**1**) exhibited structural disorder and some constraints and restraints on the phenyl rings, and nearby fragments. In particular, the phenyl bound to C32, C33, C14 and C34, were each characterized by two images that were refined with 0.62/0.38 (C103/C10b, C104/C10c and C44/C44a) and 0.51/0.49 (C102/C10a) site occupancy factors. During refinement, all the aromatic rings were modelled with constraints that assumed the presence of an idealized hexagon. Additional restraints were required to avoid the presence of adjacent carbon having too different anisotropic displacement parameters. The restraints were applied on five phenyl rings having the following carbon atoms C43, C44, C10c, C102, C10b. For the same disordered fragments, we applied distance restraints on the bonds connecting the phenyl ring with the carbonyl groups.

Powder X-ray diffraction (PXRD). PXRD measurements were undertaken in a Shimadzu XRD7000 Maxima diffractometer, operating with Cu K-α radiation at 40 kV and 30 mA. Measurements were carried out over an angular range of 5°≤ 2θ ≤ 80° with a scanning step of 0.02 and integration time of 0.60 s. Divergence, scattered and receiving radiation slits were 1°, 1° and 0.3 mm, respectively.

Elemental Analyzes (CHN). CHN elemental analyses were performed using a PerkinElmer 2400 Series II at the Analytical Center of the Institute of Chemistry, University of São Paulo (USP), São Paulo, Brazil.

Fourier-transform infrared spectroscopy (FTIR). FTIR spectra were obtained using a Perkin Elmer spectrometer of Frontier model, equipped with ATR diamond. The measurements were undertaken from 4000 cm^{-1} to 400 cm^{-1}, with 2 cm^{-1} of resolution and 120 scans.

Thermogravimetry (TG). Thermogravimetry of the complexes was performed in a TGA Q500 V20.13, from 25 °C to 950 °C, with a heating rate of 10 °C min^{-1}, and an atmosphere of air (40 mL min^{-1})/N_2 (60 mL min^{-1}).

Differential scanning calorimetry (DSC): DSC was collected for the crystals in a Q10 - TA instruments from -80 °C to 180 °C (heating ramp of 20 °C min^{-1}), using a closed alumina crucible, and a gas atmosphere of N_2 (100 mL min^{-1}).

Diffuse reflectance spectroscopy (DRS). DRS was recorded using a Shimadzu UV-2600 spectrometer equipped with solid-state and liquid sample holders. The solid-state integrating sphere is coated with $BaSO_4$, which also serves as reference for DRS measurements. The spectral resolution was set to 1 nm.

Room Temperature Photoluminescence (**PL**). PL were carried out in a Fluorolog-3 spectrofluorometer (Horiba FL3-22-iHR320) with double-gratings (1,200 grooves mm^{-1}, 330 nm blaze) in the excitation monochromator and double-gratings (1200 grooves mm^{-1}, 500 nm blaze) in the emission monochromator. An ozone-free xenon lamp of 450 W (Ushio) was used as a radiation source. A 150 W pulsed xenon lamp was used for time-resolved measurements by using a time-correlated single-photon counting (TCSPC) system. A photomultiplier (Hamamatsu R928P) operating at 950 V was used to collect the spectra in the ultraviolet and visible spectral regions, which were corrected according to the optical system of the emission monochromator and the photomultiplier response. The emission and excitation spectra were carried using the front face mode at 22.51° for the solid crystals and PMMA films or in the right angle for the chloroform solutions. The excitation and emission slits were kept in a position to lead to a spectral bandpass of 1 nm in all experiments.

Temperature-dependent photoluminescence (**PL**): Emission spectra from 77 K to 440 K were measured in the previously mentioned Fluorolog 3 equipment. The excitation and emission slits were kept in a position to lead to a spectral bandpass of 1 nm in all experiments. To control the temperature, a Linkam accessory (THMS600), with an optical fiber set (NA = 0.22 - Horiba-FL-3000/FM4-3000) was used.

Absolute emission quantum yield. Photoluminescence quantum yield (PLQY) was measured in a Quanta Phi-2 integrating sphere coupled to the previously mentioned fluorimeter. For reference, the empty sphere coated with Spectralon (reflectance > 95%) was used.

Supplementary note S2 – Data analysis

Photophysical properties of Eu^{III}. The radiative decay rate (A_{rad}) of the Eu^{III} $^5D_0$ level was calculated from eqn S1 and S2, where A is the Einstein coefficient of spontaneous emission. In eqn S1, the term $I_{0\text{-}\lambda}$ is the area under the curve related to the $^5D_0 \rightarrow ^7F_\lambda$ transition, $h\nu_{0-\lambda}$ is the energetic barycenter of the 0–λ transition, and $A_{01} = 14.65\ n^3$ in s^{-1}, where n is the refractive index (complex = 1.5, chloroform = 1.445;[6] PMMA = 1.490[7]). The non-radiative decay rate (A_{nrad}) of the Eu^{III} $^5D_0$ level was calculated from eqn S3, where τ is the $^5D_0$ level lifetime. Finally, the intrinsic Eu^{III} emission quantum yield (Q_{Eu}^{Eu}) was determined from eqn S4. The average lifetime was calculated from equation S5, where the term A_i is the pre-exponential factor, or amplitude associate to each lifetime (τ_i) values.[8]

$$A_{0-\lambda} = A_{01}\frac{I_{0-\lambda}}{I_{0-1}}\frac{h\nu_{0-1}}{h\nu_{0-\lambda}} \quad (S1)$$

$$A_{rad} = \sum_J A_{0-J} \quad (S2)$$

$$A_{total} = \frac{1}{\langle\tau\rangle} = A_{rad} + A_{nrad} \quad (S3)$$

$$Q_{Eu}^{Eu} = \frac{A_{rad}}{A_{rad} + A_{nrad}} \quad (S4)$$

$$\langle\tau\rangle = \frac{\sum A_i \tau_i^2}{\sum A_i \tau_i} \quad (S5)$$

The experimental Judd-Ofelt intensity parameters were calculated from eqn S6, where $|\langle ^7F_J \| U^{(\lambda)} \| ^5D_0 \rangle|^2$ represents the square reduced matrix elements, which values are equal to 0.0032 for Ω_2 and 0.0023 for Ω_4; h is the Planck's constant, e is the electron charge, c is the speed of light in vacuum, ω *is the* angular frequency of the incident radiation field, ε_0 is vacuum permittivity constants, and X is the Lorentz local field correction equal to $n(n^2+2)^2/9$.[9]

$$\Omega_\lambda = \frac{3hc^3 A_{0\lambda}}{8\pi e^2 \omega^3 \chi |\langle ^7F_J \| U^{(\lambda)} \| ^5D_0 \rangle|^2} \quad (S6)$$

Luminescence thermometry. The relative thermal sensitivity of the complexes was calculated from eqn S6, where Δ is the thermometric parameter and T is the temperature. The temperature uncertainty (δT) is calculated by equation S7, where $\delta\Delta/\Delta$ is the relative uncertainty in the determination of the $^5D_0$ lifetime, which was considered as at about 0.02 ms.[10]

$$S_r = \frac{1}{\Delta}\left|\frac{d\Delta}{dT}\right| \quad (S7)$$

$$\delta T = \frac{1}{S_r}\frac{\delta\Delta}{\Delta} \quad (S8)$$

Supplementary note S3 – Crystallographic data

Table S1. Crystallographic data of the $[Eu_2(bpm)(dbm)_6]$ (**1**) and $[Eu_2(bpm)(btfa)_6]$ (**2**) complexes.

	**1**	**2**
CCDC deposition number	2480172	2480173
Empirical formula	$C_{98}H_{72}Eu_2N_4O_{12}$	$C_{68}H_{42}Eu_2F_{18}N_4O_{12}$
Molecular weight, g mol^{-1}	1801.51	1752.97
Temperature, K	200	200
Wavelength, Å	0.71073	0.71073
Crystal system	Monoclinic	Triclinic
Space group	$P2_1/n$	$P\bar{1}$
a, Å	13.309(4)	9.7256(3)
b, Å	14.468(6)	11.0002(4)
c, Å	21.950(5)	17.0092(6)
α, °	90	72.706(1)
β, °	102.804(12)	80.215(1)
γ, °	90	84.304(1)
Volume	4122(2)	1709.87(10)
No. of formula units/unit cell, Z	2	1
Density (*ρ*), g cm^{-3} calc'd.	1.452	1.702
μ / mm^{-1}	1.57	1.93
F(0 0 0)	1820.0	862
Crystal size, mm^3	0.08 × 0.05 × 0.03	0.2x0.04×0.02
Minimum and maximum transmittance	0.617, 0.745	0.589, 0.746
Theta range, °	2.163 - 21.889	2.536 - 21.282
Index ranges (h k l)	(15 17 26)	(12 14 22)
No. of reflections measured	79326	16947
No. of independent reflections	7815	8476
R(int)	0.099	0.049
Completeness, %	0.999	1.000
Data / restraints / parameters	7815 / 188/ 645	8476 / 0 / 469
R1, wR^2 (I > 2 σ(I))[a]	0.0366, 0.0697	0.0440, 0.0917
R1, wR^2 (all data)	0.0612, 0.0817	0.0555, 0.0970
Goodness of fit on F^2	1.026	1.060
Largest differential peak and hole, e$^-$/Å^3	1.10, −0.55	1.25, −1.78

Table S2. Shape analysis using SHAPE 2.1 [11] of the Eu^{III} polyhedron of in the $[Eu_2(bpm)(dbm)_6]$ (**1**) and $[Eu_2(bpm)(btfa)_6]$ (**2**). The values in the table represent the continuous shape measures (CShM, dimensionless) corresponding to each idealized geometry.

Idealized geometry	Short name	Point group	1	2
Triangular dodecahedron	TDD-8	D_{2d}	**1.152**	1.69507
Biaugmented trigonal prism	BTPR-8	C_{2v}	1.730	2.27259
Square antiprism	SAPR-8	D_{4d}	2.940	**0.85101**
Biaugmented trigonal prism J50	JBTPR-8	C_{2v}	2.253	2.86900
Snub diphenoid J84	JSD-8	D_{2d}	3.402	4.62380
Cube	CU-8	*Oh*	10.997	9.48624
Triakis tetrahedron	TT-8	*Td*	11.620	10.25350
Johnson gyrobifastigium J26	JGBF-8	D_{2d}	13.165	15.87153
Hexagonal bipyramid	HBPY-8	D_{6h}	16.505	15.97410
Heptagonal pyramid	HPY-8	C_{7v}	24.076	22.69842
Elongated trigonal bipyramid	ETBPY-8	D_{3h}	24.977	23.26243
Johnson elongated triangular bipyramid J14	JETBPY-8	D_{3h}	29.381	27.35358
Octagon	OP-8	D_{8h}	33.576	30.64350

Table S3. Eu – N and Eu – O bond distances (Å) in the first coordination sphere of $[Eu_2(bpm)(dbm)_6]$ (**1**) and $[Eu_2(bpm)(btfa)_6]$ (**2**).

Bond	1	2
**Eu – N21**	2.6587(27)	2.6372(28)
**Eu – N61**	2.7172(38)	2.6382(33)
**Eu – O12**	2.3156(31)	2.3557(29)
**Eu – O13**	2.3681(25)	2.3400(33)
**Eu – O14**	2.3867(27)	2.3492(29)
**Eu – O22**	2.3178(28)	2.3575(22)
**Eu – O23**	2.3257(32)	2.3456(25)
**Eu – O24**	2.3332(30)	2.3544(22)
**Average Eu – O**	2.341	2.350

Table S4. Bite angles (°) of ligands in $[Eu_2(bpm)(dbm)_6]$ (**1**) and $[Eu_2(bpm)(btfa)_6]$ (**2**).

Bond	1	2
**N21 – Eu – N61**	60.053(100)	61.455(92)
**O12 – Eu – O22**	71.691(96)	72.192(83)
**O14 – Eu – O24**	69.264(95)	72.238(103)
**O13 – Eu – O23**	71.984(100)	73.076(91)

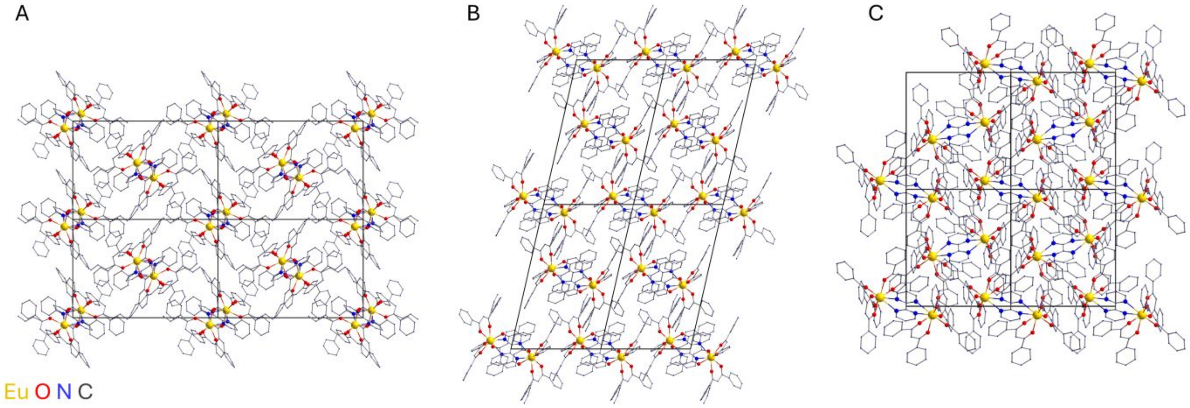

Figure S1. View of the packing arrangement along the crystallographic (a) a, (b) b, and (c) c-axis in $[Eu_2(bpm)(dbm)_6]$ (**1**).

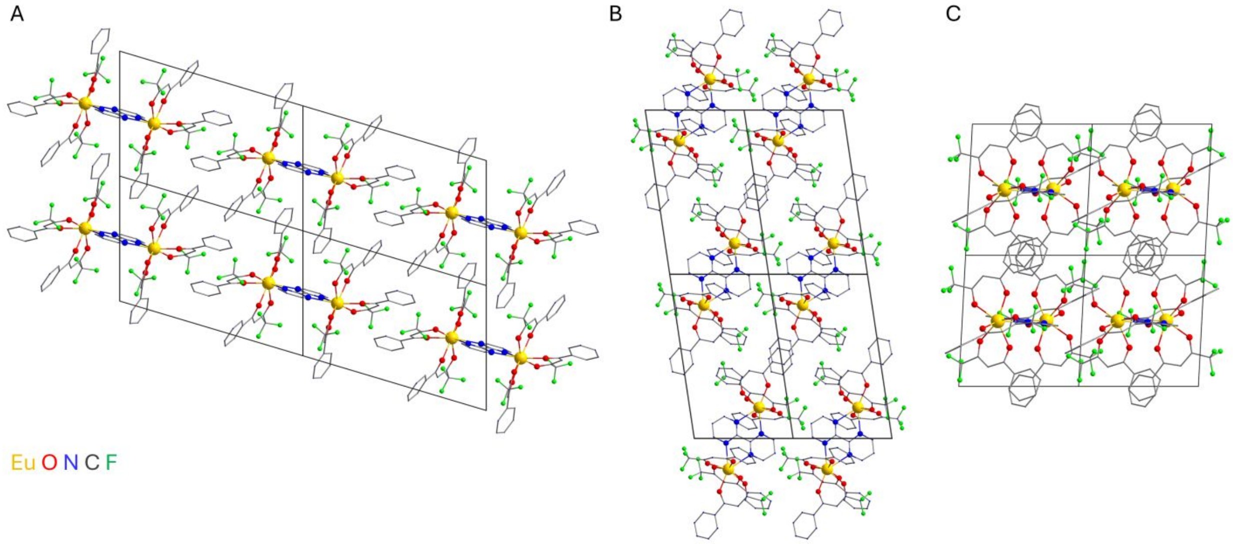

Figure S2. View of the packing arrangement along the crystallographic (a) a, (b) b, and (c) c-axis in $[Eu_2(bpm)(btfa)_6]$ (**2**).

Table S5. Shortest intermolecular and intramolecular (Å) Eu – Eu bond distances in the $[Eu_2(bpm)(dbm)_6]$ (**1**) and $[Eu_2(bpm)(btfa)_6]$ (**2**) complexes.

Bond	1	2
**Intramolecular**	6.9444(14)	6.9125(5)
**Intermolecular**	7.8994(20)	9.3227(5)

Table S6. Intermolecular hydrogen bond distances (Å) in [Eu_2(bpm)(dbm)$_6$] (**1**).

Contact	Bond distance / Å
**O13···H22**	3.081
**O13···H6**	3.048
**O14···H133**	2.640
**O13···H133**	2.778

Table S7. Intermolecular and intramolecular hydrogen bond distances (Å) in [Eu_2(bpm)(btfa)$_6$] (**2**).

Contact	Bond distance / Å
**Intramolecular**	
**F22···H63**	3.239
**H2···F13**	2.417
**H4···F34**	2.994
**H102···F13**	2.852
**Intermolecular**	
**O14 H3**	3.261
**O12 H3**	3.087
**H93 F14**	3.138
**H64 F24**	2.817
**H64 F34**	2.803
**F12 H34**	3.103
**H72 F23**	2.664
**H33 F33**	2.872
**H103 F23**	3.078
**H103 F33**	2.591

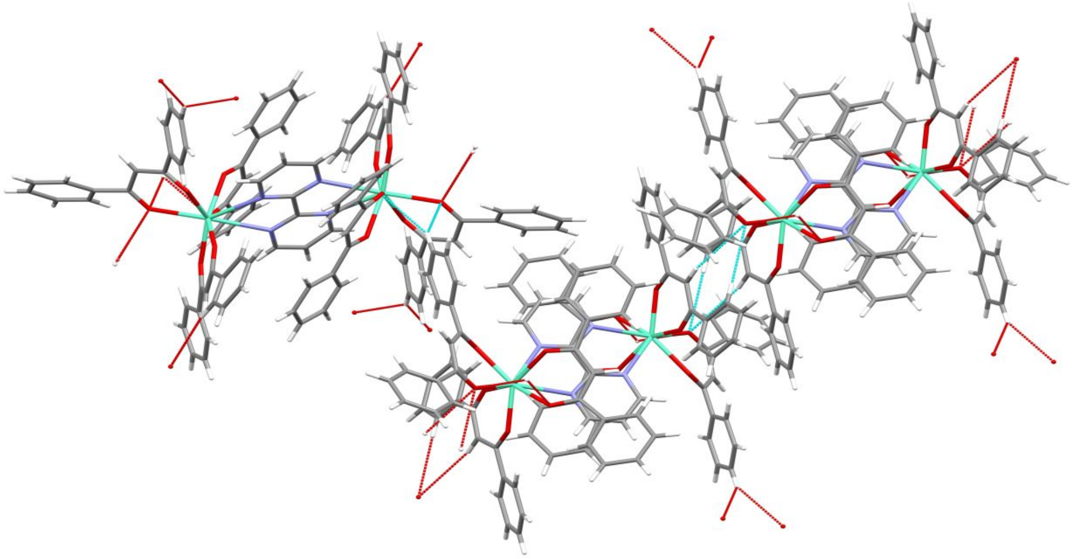

Figure S3. Representation of the intermolecular H-bonds (dashed blue and red lines) in $[Eu_2(bpm)(dbm)_6]$ (**1**). Carbon = gray; hydrogen = white; europium = cyan; oxygen = red; nitrogen = light violet.

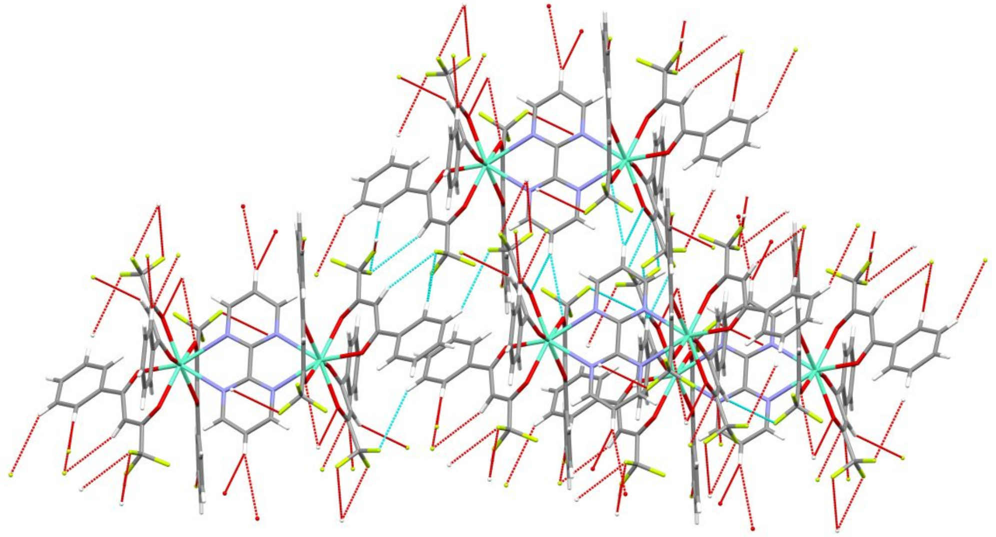

Figure S4. Representation of the intermolecular H-bonds (dashed blue and red lines) in $[Eu_2(bpm)(btfa)_6]$ (**2**). Carbon = gray; hydrogen = white; europium = cyan; oxygen = red; nitrogen = light violet; fluorine = green.

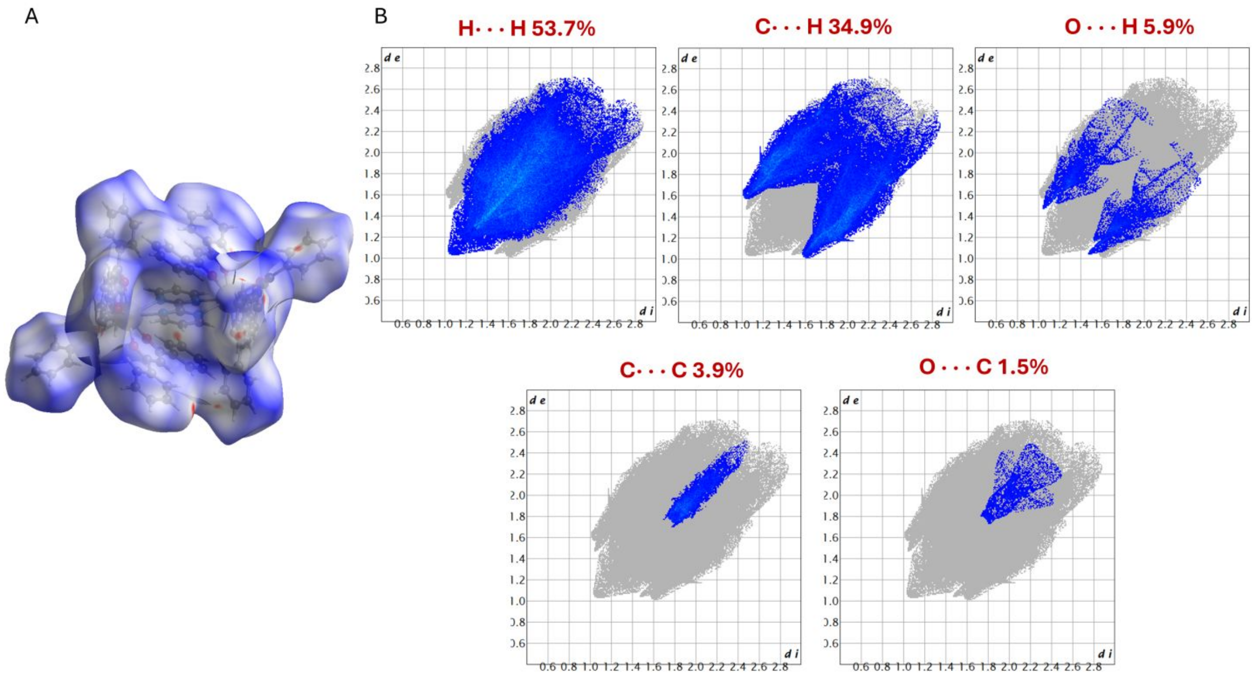

Figure S5. (a) Hirshfeld surface (HS) of [Eu_2(bpm)(dbm)$_6$] (**1**) mapped over d_{norm} and shape index, *S*. In the d_{norm} HS, a red–blue–white colour scheme was used, whereas red regions represent closer contacts, blue regions represent longer ones, and white regions represent the distance of contacts which is exactly equal to the *vdW* separation. (b) The 2D fingerprint plots of interatomic interactions of [Eu_2(bpm)(dbm)$_6$], showing the percentages of contacts that contribute to the total Hirshfeld surface area of the molecules.

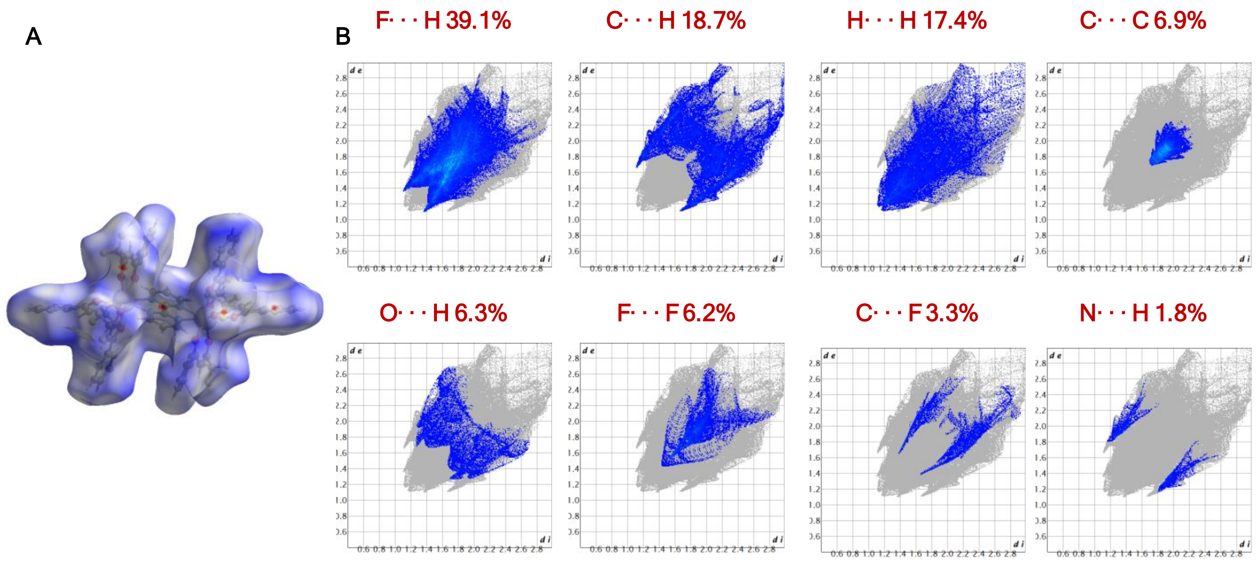

Figure S6. (a) Hirshfeld surface (HS) of [Eu_2(bpm)(btfa)$_6$] (**2**) mapped over d_{norm} and shape index, *S*. In the d_{norm} HS, a red–blue–white colour scheme was used, whereas red regions represent closer contacts, blue regions represent longer ones, and white regions represent the distance of contacts which is exactly equal to the *vdW* separation. (b) The 2D fingerprint plots of interatomic interactions of [Eu_2(bpm)(dbm)$_6$], showing the percentages of contacts that contribute to the total Hirshfeld surface area of the molecules.

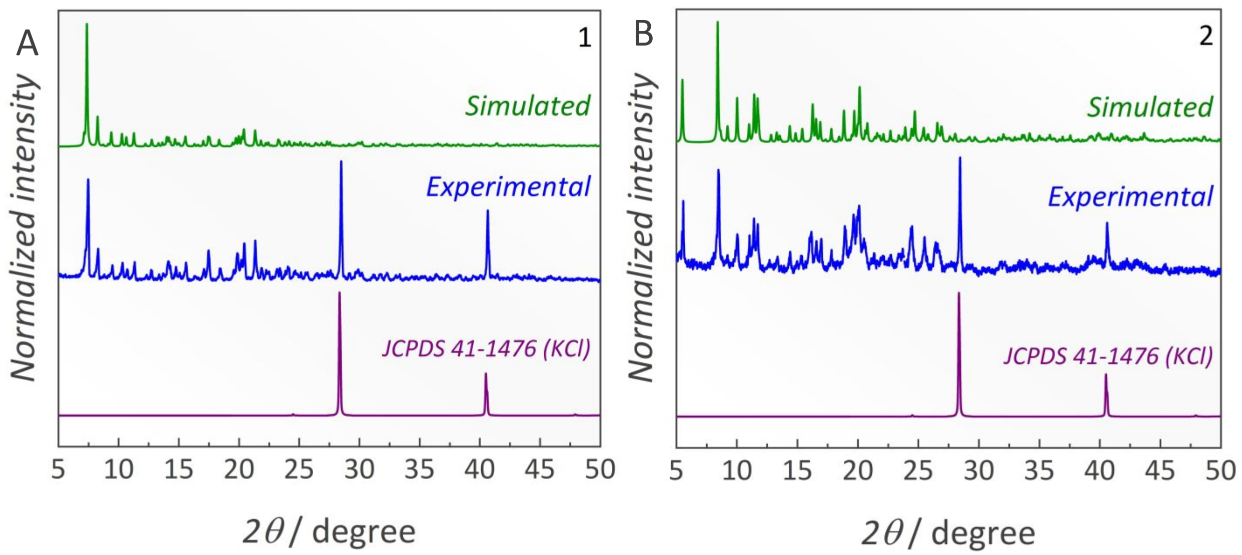

Figure S7. Powder XRD of (a) [Eu_2(bpm)(dbm)$_6$] (**1**) and (b) [Eu_2(bpm)(btfa)$_6$] (**2**) compared to the simulated PXRD pattern determined from the SC-XRD of the respective dinuclear complexes and the JCPDS card 41-1476 (cubic KCl phase). KCl is a subproduct of crystallization and arises from KOH used as base to deprotonate the ligand and $EuCl_3$ used as precursor. KCl is optically inert and does not compromise the spectroscopic analyses.

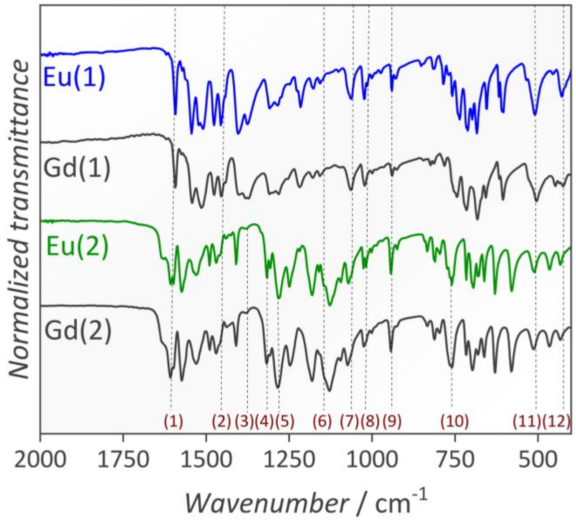

	Wavenumber / cm^{-1}				
	**1-Eu**	**1-Gd**	**2-Eu**	**2-Gd**	
(1)	1591	1593	1598	1598	ν(C=O)
(2)	1455	1455	1458	1458	ν ar, C-C
(3)	-	-	1381	1377	ν(C-F)
(4)	-	-	1317	1318	$\nu(CF_3)$
(5)	-	-	1282	1284	ν(C–F)
(6)	1146	1146	1144	1144	δ_{ip} ar C-H
(7)	1061	1064	1060	1060	δ_{ip} ar C-H
(8)	1023	1021	1024	1024	δ_{ip} ar C-H
(9)	940	938	942	941	(δ_{oop} C-H)
(10)	-	-	759	578	δ(C-F)
(11)	507	504	509	511	Δ + δCC ϕ
(12)	427	434	431	432	νO-Eu

Figure S8. FTIR spectra of $[Ln_2(bpm)(dbm)_6]$ (**1**) and $[Ln_2(bpm)(btfa)_6]$ (**2**) (Ln = Eu^{III} or Gd^{III}) complexes as crystals measured at 300 K. The corresponding spectral assignments are provided in the adjacent table.

Supplementary note S4 – Thermogravimetry and differential scanning calorimetry

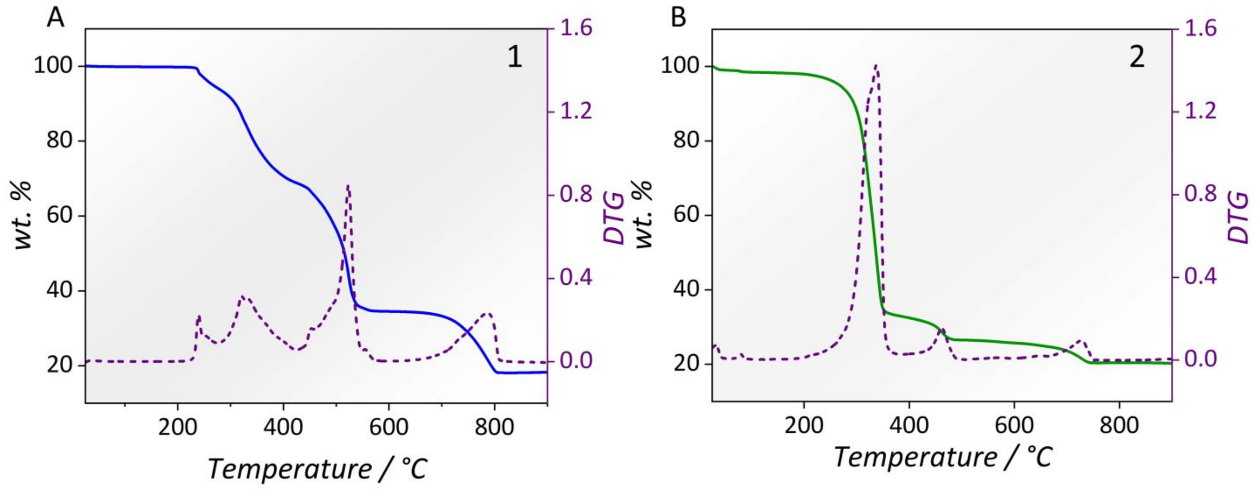

Figure S9. Thermogravimetry (TG) and derivative thermogravimetry (DTG) of (a) $[Eu_2(bpm)(dbm)_6]$ (**1**) and (b) $[Eu_2(bpm)(btfa)_6]$ (**2**) as crystals.

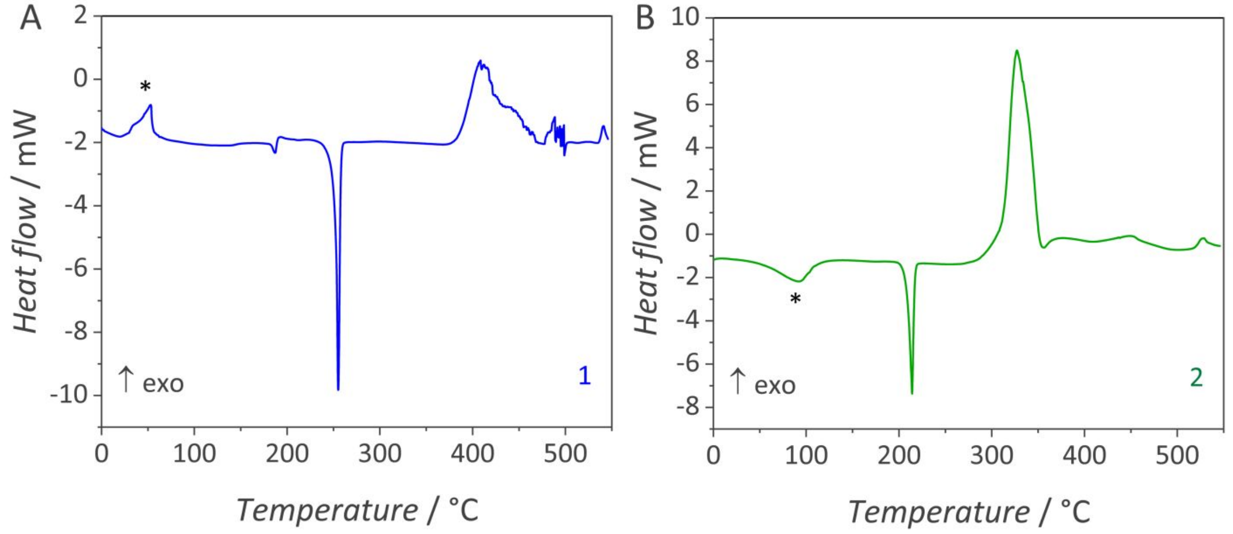

Figure S10. Differential scanning calorimetry (DSC) measurements of (a) $[Eu_2(bpm)(dbm)_6]$ (**1**) and (b) $[Eu_2(bpm)(btfa)_6]$ (**2**) as crystals.* Adsorbed solvent release.

Supplementary note S5 – Additional spectroscopic data

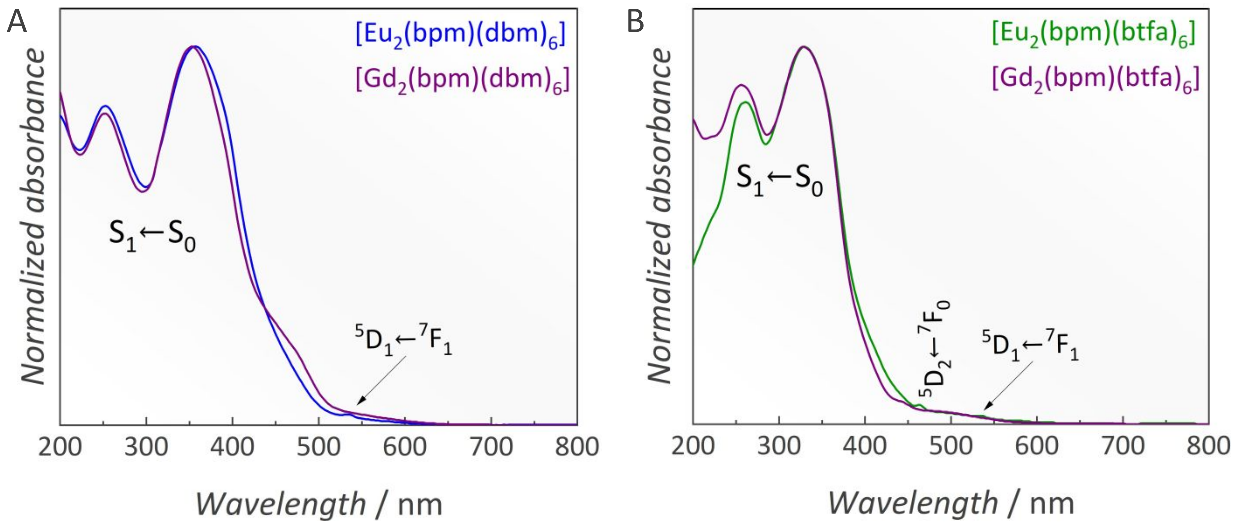

Figure S11. Diffuse reflectance spectra (DRS) of (a) $[Ln_2(bpm)(dbm)_6]$ and (b) $[Ln_2(bpm)(btfa)_6]$ (Ln = Eu^{III} or Gd^{III}) complexes measured for the crashed crystals at 300 K.

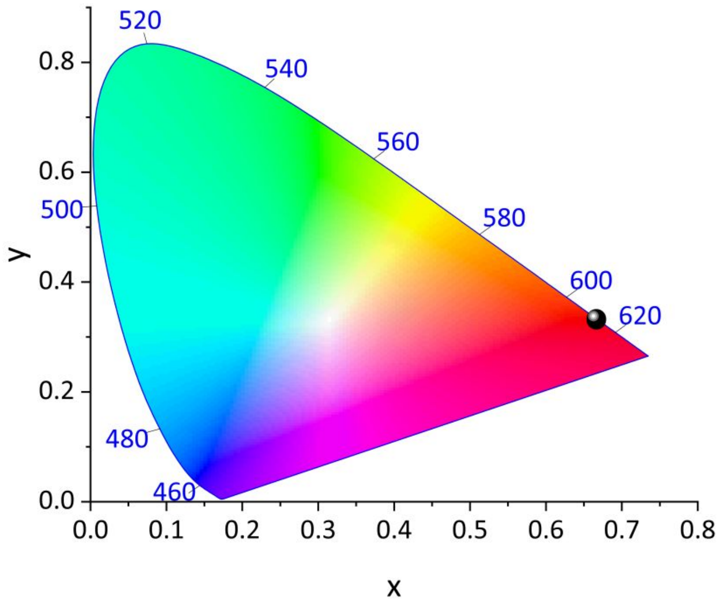

Figure S12. CIE (*Commission Internationale de l'éclairage*) chromaticity diagram calculated from the emission spectra of $[Eu_2(bpm)(dbm)_6]$ (**1**) and $[Eu_2(bpm)(btfa)_6]$ (**2**) as crystals**,** both represented by overlapping black circles. The CIE coordinates of **1** and **2** are (0.666;0.333) and (0.666;0.332), respectively.

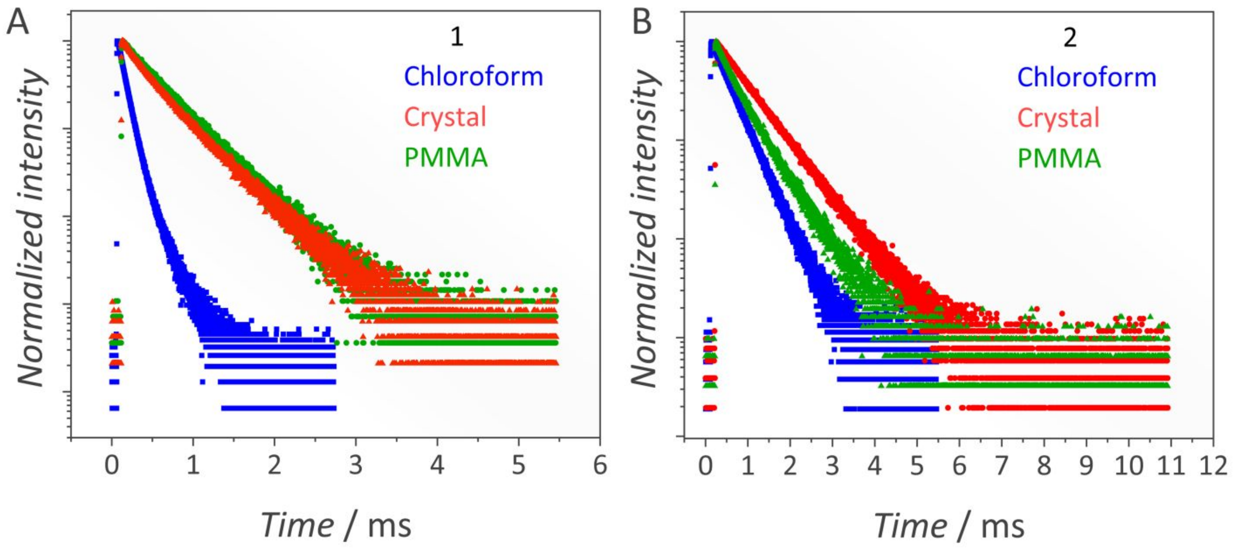

Figure S13. Emission decay curves (300 K) of [Eu_2(bpm)(dbm)$_6$] (**1**) monitored at (a) λ_{exc} = 330 nm, λ_{em} = 612 nm and (b) [Eu_2(bpm)(btfa)$_6$] (**2**) monitored at λ_{exc} = 370 nm, λ_{em} = 612 nm. The decay curves were undertaken for the complex as crystals, chloroform solution (1 mg mL^{-1}), or PMMA films (1wt%).

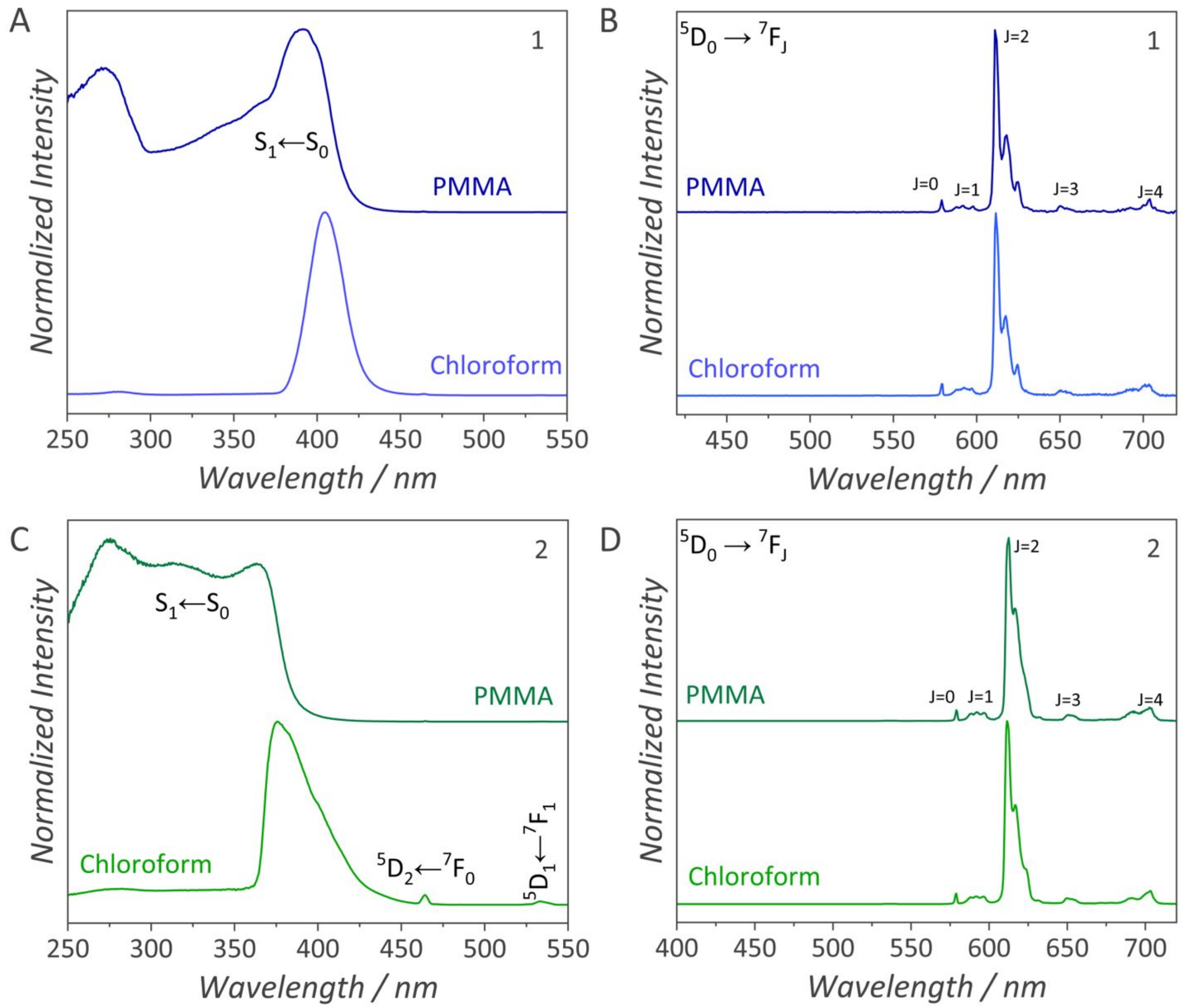

Figure S14. (a) Excitation (λ_{em}= 612 nm) and (b) emission spectra (λ_{ex}= 405 nm) spectra of $[Eu_2(bpm)(dbm)_6]$ (**1**) in chloroform solution (1 mg mL^{-1}) or PMMA films (1wt.%). (c) Excitation (λ_{em}= 612 nm) and (d) emission spectra (λ_{ex}= 370 nm) spectra of $[Eu_2(bpm)(btfa)_6]$ (**2**) in chloroform solution (1 mg mL^{-1}) or PMMA films (1wt.%).

Table S8. $^5D_0$ level lifetime (τ) as well as radiative (A_{rad}) and non-radiative (A_{nrad}) decay rates determined for [Eu_2(bpm)(dbm)$_6$] (**1**) and [Eu_2(bpm)(btfa)$_6$] (**2**), in the solid state, in chloroform solution (1 mg mL^{-1}), or in PMMA films (1wt.%).

	Complex	τ / ms	A_{rad} / s^{-1}	A_{nrad} / s^{-1}
**Crystal**	**1**	0.435	1091.36	1204.33
	**2**	0.753	602.83	725.19
**Chloroform**	**1**	0.106	862.56	8580.31
	**2**	0.439	950.96	1325.90
**PMMA film**	**1**	0.402	1203.27	1283.05
	**2**	0.536	1047.09	817.88

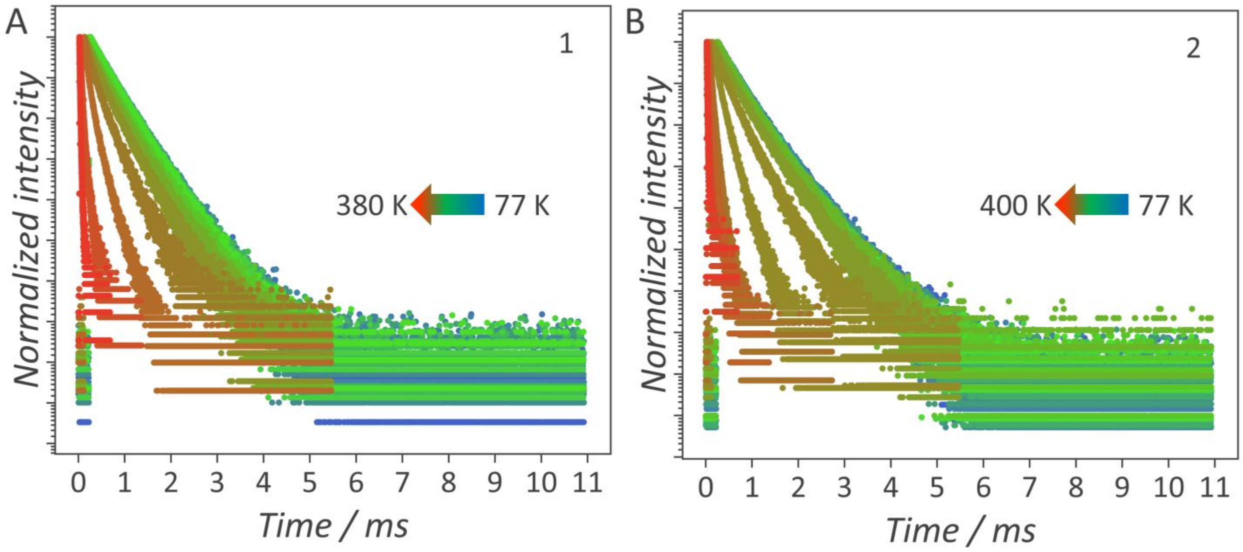

Figure S15. Temperature-dependent emission decay curves of the PMMA films (1wt.%) for (a) [Eu_2(bpm)(dbm)$_6$] (**1**) monitored at λ_{exc} = 330 nm, λ_{em} = 612 nm or (b) [Eu_2(bpm)(btfa)$_6$] (**2**) monitored at λ_{exc} = 370 nm, λ_{em} = 612 nm.

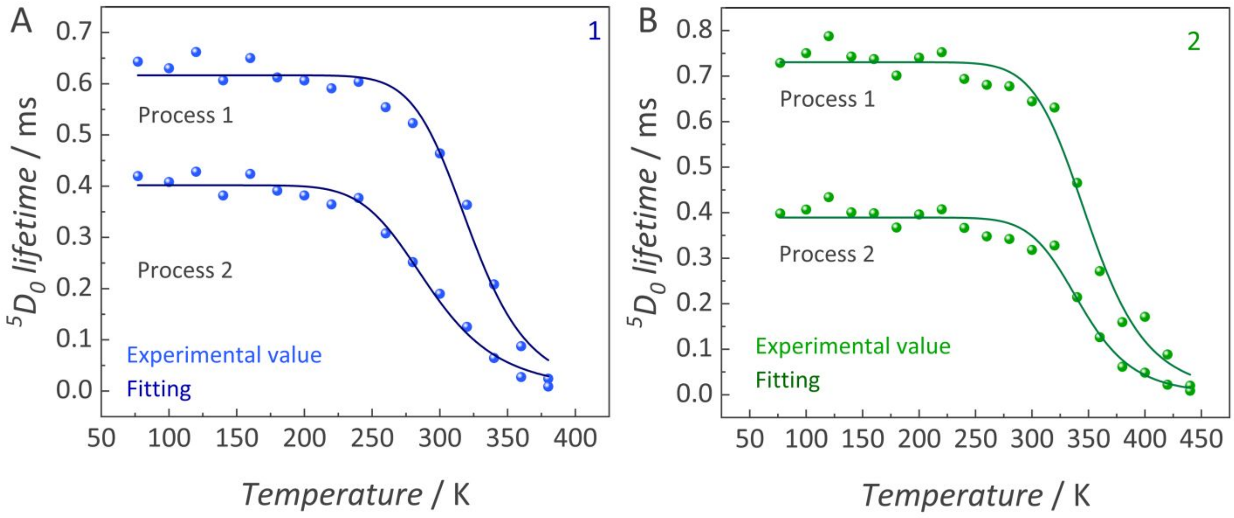

Figure S16. Temperature dependence of the $^5D_0$ lifetime (τ_1 and τ_2, determined from a biexponential adjustment of the emission decay curves, Figure S15) and best fits to the Mott-Seitz function for (a) $[Eu_2(bpm)(dbm)_6]$ (**1**) and (b) $[Eu_2(bpm)(dbm)_6]$ (**2**) as PMMA films (1wt.%); fitting parameters are listed in Table 2.

Supplementary references

[1] Errulat, D.; Gabidullin, B.; Murugesu, M.; Hemmer, E. Probing Optical Anisotropy and Polymorph-Dependent Photoluminescence in [Ln2] Complexes by Hyperspectral Imaging on Single Crystals, *Chem. Eur. J.* 2018, 24, 40, 10146- 10155.

[2] Beltrame, A. C. F.; Bispo-Jr, A. G.; Canisares, F. S. M.; Fernandes, R. V.; Laureto, E.; Lima, S. A. M.; Pires A. M. PMMA or PVDF films blended with β-diketonate tetrakis Eu^{III} or Tb^{III} complexes used as downshifting coatings of near-UV LEDs, Soft Matter 2023, 19, 3992 – 4000.

[3] Sheldrick, G. M. SADABS-2008/1 - Bruker AXS Area Detector Scaling and Absorption Correction Bruker AXS: Madison, Wisconsin, USA, 2008.

[4] Sheldrick, G. M. *Acta Crystallogr A* 2015, 71, 3–8.

[5] Dolomanov, O. V.; Bourhis, L. J.; Gildea, R. J.; Howard, J. A. K.; Puschmann, H. *J Appl Crystallogr* 2009, 42, 339–341.

[6] Bhat, S. A.; Iftikhar, K. Optical properties and intensity parameters of UV excited efficient red emitting europium complexes containing fluorinated 1, 3-dione as primary sensitizer in solution, solid and PMMA thin films, *Opt. Mater.* 2020, 99 109600.

[7] Moura Jr, R. T.; Quintano, M.; Santos Jr, C. V.; Albuquerque, V. A. C. A.; Aguiar, E. C.; Kraka, E.; Neto, A. N. C. Featuring a new computational protocol for the estimation of intensity and overall quantum yield in lanthanide chelates with applications to Eu (III) mercapto-triazole Schiff base ligands, Opt. Mat 2022 16, 100216.

[8] Ma Y.; Wang, H.; Liu, W.; Wang, Q.; Xu, J.; Tang, Y. Microstructure, Luminescence, and Stability of a Europium Complex Covalently Bonded to an Attapulgite Clay, *J. Chem. Phys. B,* 2009,113,43, 14139-14145.

[9] C. Kodaira, H. F. Brito, O. L.Malta, O. A. Serra, *J. Lumin. 2003*, **101**, 11.

[10] Brites, C. D. S.; Millán, A.; Carlos, L. D. Lanthanides in Luminescent Thermometry. In: Handbook on the physics and chemistry of rare earths. Elsevier 2016, 49, 339-427.

[11] Pinsky, M.; Avnir, D. Continuous symmetry measures. 5. The classical polyhedral, *Inorg. Chem.* 1998, 37, 21, 5575-5582.
